# Geometrically Encoded Positioning of Introns, Intergenic Segments, and Exons in the Human Genome

**DOI:** 10.1002/advs.202509964

**Published:** 2025-10-27

**Authors:** Luay M. Almassalha, Kyle L. MacQuarrie, Marcelo Carignano, Cody L. Dunton, Ruyi Gong, Joe Ibarra, Lucas M. Carter, Wing Shun Li, Rikkert Nap, Parambir S. Dulai, Igal Szleifer, Vadim Backman

**Affiliations:** ^1^ Division of Gastroenterology and Hepatology Northwestern Memorial Hospital Chicago IL 60611 USA; ^2^ Center for Physical Genomics and Engineering Northwestern University Evanston IL 60208 USA; ^3^ Stanley Manne Children's Research Institute Ann and Robert H. Lurie Children's Hospital of Chicago Chicago IL 60611 USA; ^4^ Northwestern University Feinberg School of Medicine Chicago IL 60611 USA; ^5^ Department of Biomedical Engineering Northwestern University Evanston IL 60208 USA; ^6^ IBIS Interdisciplinary Biological Sciences Graduate Program Northwestern University Evanston IL 60208 USA; ^7^ Applied Physics Program Northwestern University Evanston IL 60208 USA; ^8^ Center for Human Immunobiology Chicago IL 60611 USA; ^9^ Department of Chemistry Northwestern University Evanston IL 60208 USA

**Keywords:** biophysics, cancer, chromatin, evolution, self‐assembly

## Abstract

Human tissues require a mechanism to generate durable, yet modifiable, transcriptional memories to sustain cell function across a lifetime. Previously, it was demonstrated that nanoscale packing domains couple heterochromatin (cores) and euchromatin (outer zone) into unified reaction volumes that can generate transcriptional memory. In prior work, this framework demonstrates that RNA synthesis occurred within the ideal zone (intermediate density) portions of the domain. Naturally, this creates a question of where genes are positioned in relation to the packing domain architecture and which genetic material fills the domain core to sustain transcription. Here, it is proposed that this can be solved by the encoded positioning of introns, intergenic segments, and exons as a projection of the functional packing layers of domains. This suggests that introns and intergenic segments are coupled to adjacent exons to generate coherent packing domain volumes. How this organization will reconcile contradictions in epigenetic patterns, non‐randomness in oncogenic mutations, and produce durable transcriptional memory is illustrated. The study concludes by showing that this genome geometry may have coincided with the rapid evolution of body‐plan complexity, suggesting that chromatin geometry could be fundamental to metazoan evolution.

## Graphical abstract

This study introduces a new hypothesis: exons, introns, and intergenic segments are non‐random projections of the functional layers of 3D structure of chromatin packing domains. Evidence is presented that this “geometric code” may encode volumetric structure, reconciling epigenetic patterns, correlates with oncogenic mutations, acting as a potential mechanism to generate transcriptional memory, and coincides with the evolution of complex body plans.

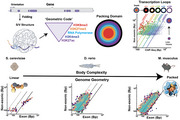

## 1 Introduction

Although cells in a multicellular organism share the same genome, they differentiate into hundreds of cell types. Even cells of the same lineage can perform different functions, shifting over timescales from minutes to decades, while many disease states arise from altered gene expression. A key underlying question is how genetic information gives rise to diverse and dynamic cell phenotypes across development, aging, and disease. Chromatin, DNA and its associated binding proteins, is frequently invoked as the mechanism by which these processes occur. Many sequence‐dependent chromatin‐transcriptional regulatory elements are well studied, including enhancers, promoters, insulators, silencers, etc.^[^ ^1^, ^2^, ^3^, ^4^ ^]^ These provide significant insight into transcriptional regulation. Paired with imaging and multi‐omics data, an emerging view is that chromatin organizes into packed structures throughout the nucleus. To measure physical structures at the nanoscale, electron microscopy (EM) has long been viewed as the gold‐standard. Earlier EM studies of chromatin organization were limited by the non‐specific binding of contrast agents. Despite these limits, it was found that chromatin organizes into areas of high density (typically the nuclear periphery) and low density (typically the nuclear interior). These findings correlated with observations on diffraction limited microscopy methods and, with the expansion of molecular mapping, helped form the current understanding of chromatin partitioning into distinct territories. In the current framework, chromatin organizes into active (A‐, nuclear interior) and inactive (B‐, nuclear periphery) micrometer sized (megabasepair) compartments. The mechanisms proposed to generate this organization has evolved over time, with several prominent models including hierarchical assembly, the fractal globule, loop extrusion, and liquid‐liquid phase separation (LLPS).

However, even though B‐ compartments enrich in heterochromatin markers (H3K9me3, H3K27me3) associated with decreased transcription and A‐ compartments the inverse (H3K4me3, H3K27ac, RNA polymerase), neither compartment is devoid of the reciprocal markers. Indeed, active RNA synthesis is observed in B‐ compartments and the nuclear periphery.^[^ ^5^, ^6^, ^7^, ^8^ ^]^ These findings have potential clinical implications central to the question of how diverse cell states arise, are maintained, and disrupted in human health. Clinically relevant models of neuronal despecialization demonstrated heterochromatin loss does not necessarily result in transcriptional activation, instead producing a paradoxical further impairment.^[^ ^7^ ^]^ Similarly, transcriptional plasticity in ovarian cancer stem cells appears dependent on heterochromatin deposition within introns and intergenic segments.^[^ ^6^ ^]^ Within the existing framework of chromatin structure‐function, it is challenging to explain these observations. A potential solution to these paradoxes may lie in the limitations of older chromatin imaging technologies now being addressed by the advent of ChromEM/ChromSTEM technologies.

On ChromEM imaging, the measured contrast is proportional to DNA density, allowing recovery of ground‐truth information of chromatin packing at length‐scales ranging from 2 nm (a few basepairs) to several microns (megabase pairs). In comparison to prior EM methods, ChromEM demonstrated chromatin transitions from the beads‐on‐a‐string into a disordered 5–20 nm chain and then into 3‐D packing domain (PD) volumes with a distribution of sizes and packing efficiencies.^[^ ^9^, ^10^, ^11^ ^]^ A striking observation of PDs is that high‐ and low‐density is coupled within unified reaction volumes, suggesting heterochromatin and euchromatin are packed together. Since ChromSTEM lacks sequence‐specific information, it was necessary to pair ChromEM modalities with molecularly specific imaging modalities (super resolution imaging), molecular technologies (Hi‐C, ChIP‐Seq, RNA‐Seq, ATAC‐seq, etc), and multi‐scale modeling. Using this integrated approach, it was confirmed that the PDs observed on ChromSTEM imaging represent unified volumes pairing heterochromatin and euchromatin together.^[^ ^8^, ^12^ ^]^ Mechanistic studies indicated PDs can self‐assemble through the interplay between transcription, cohesin, and nucleosome remodeling enzymes. One can consider self‐assembly in complement to prior observations of LLPS condensates and loop extrusion. LLPS and loop extrusion have offered explanations for the observed sizes of nanoscopic domains and the preferential enrichment of heterochromatin and euchromatin within them. For example, in LLPS models, the rate of separation into heterochromatic segments depends on factors regulating the underlying reactions on the nucleosomes themselves, including enzyme concentrations, molecular crowding, and chromatin density—which shift the balance toward heterochromatin condensate expansion that is limited by acetylation at the exterior.^[^ ^13^, ^14^, ^15^ ^]^ In loop extrusion, the mechanical activity of cohesins, condensins, and other motor proteins are proposed to act as a topological barrier to prevent expansion, resulting in the discretization of the genome. A limitation of current LLPS or loop extrusion models is the inability to explain how heterochromatin and euchromatin appear to occupy shared volumes now observable with the advent of ChromSTEM imaging as a unified physical structure regulating gene transcription.

PD self‐assembly builds on the understanding of loop extrusion and LLPS while introducing a mechanism for transcriptionally mediated cellular memory encoded in the generated volumes.^[^ ^12^, ^16^ ^]^ This occurs because the act of transcription generates loops (forced returns or “loopons”) that create a local continuous density gradient that geometrically guides nucleosome remodeling enzymes based on size (heterochromatin enzymes ≈2 nm, euchromatin ≈6 nm).^[^ ^10^ ^]^ This results in deposition of heterochromatin tightly within the domain core that gradually decreases outward. The gradual decay produces an intermediate “ideal” zone for optimal positioning of RNA polymerase II (Pol‐II) and transcription factors. Paired with modeling and super resolution imaging, we showed that this efficient packing – heterochromatin deposition into cores – acts to increase transcriptional output at ideal density zones.^[^ ^12^ ^]^ It is through this mechanism that a signaling process can produce a “transcriptional memory” that is sustained even after the initial transcriptional signal resolved. Even though transcription appears to favor the intermediate “ideal” region, the question emerges of where the information for geometric system is stored in the human genome.

**Figure 1:**
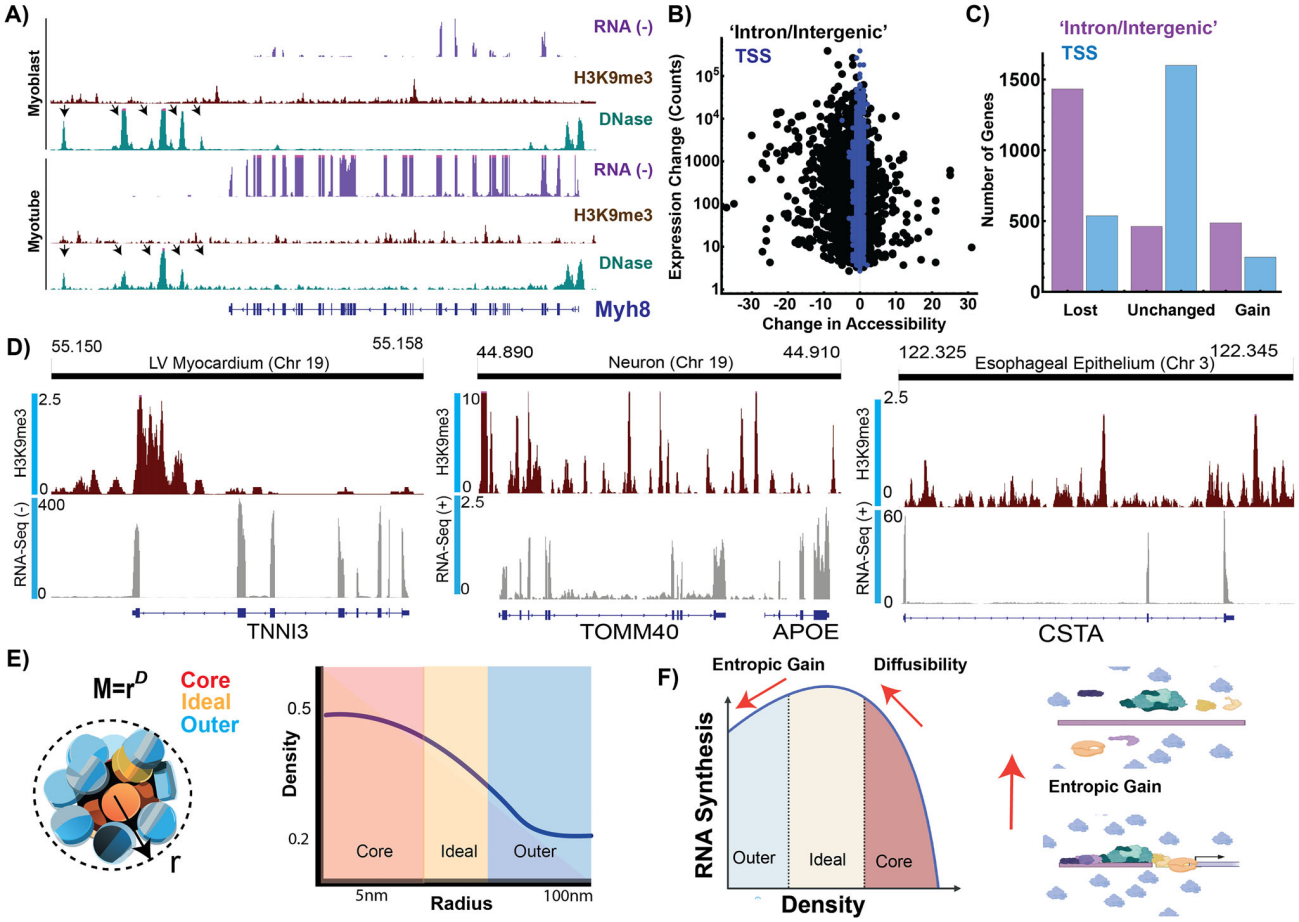
A paradox of the loss of accessibility with transcriptional amplification. A) DNAse‐seq analysis demonstrates loss of accessibility with concurrent transcriptional activation during muscle differentiation. B,C) Loss of accessibility in non‐exonic segments occurs across myogenic transcriptional activation (n = 2381 statistically significantly differentially activated genes, 60.1% of genes lose at least 1 ATAC‐seq peak; 95% confidence interval of 58–62%). D) Transcriptional activity is associated with intronic heterochromatin deposition across tissue types. These are biallelic, constitutively expressed genes in their respective tissues. Critically, this includes TNNI3 (troponin), which is a component of the sarcomere necessary for cardiac contractility. E) Mass‐fractal packing domains create a unified reaction‐volume due to the continuous density gradient across domain layers. F) Transcription is non‐monotonically dependent on local density due to the trade‐off between entropic gain (remaining bound as an intermediate complex decreases the excluded volume to other macromolecules) and the diffusibility of reactant species. As density continues to increase, Pol II subunits can no longer penetrate deeper into a domain volume resulting in inhibition.

We applied these principles of PD self‐assembly to muscle differentiation, observing that the activation of transcription on exons on the genes Myh1 and Myh2 was associated with the deposition of heterochromatin within non‐exonic (NE) segments (introns, intergenic segments) of gene bodies.^[^ ^12^ ^]^ Naturally, we wondered if this observation provides a geometric system for genes to pack into nanoscale structures: NE elements act to produce the PD volumes that could position exons into the ideal zones. Using myogenesis again as a model system, to our surprise, we observed that 60% of differentially activated genes (log fold 2 >1, adjusted p‐value <0.05; n = 2381) in muscle differentiation are associated with decreased accessibility in NE segments (**Figure** **1**a‐c). This behavior contrasts with accessibility at the transcription start site, which is generally stable (Figure 1b,c). Interestingly, transcriptionally stable genes (‐0.25< log fold 2 <0.25, adjusted p‐value >0.5; n = 2350) demonstrated loss of accessibility within NE segments but not the degree observed in transcriptionally activated genes (Figure S1, Supporting Information). Given this finding, we then examined whether heterochromatin resides in NE segments with adjacent RNA synthesis across human tissues broadly by analyzing data available through ENCODE.^[^ ^17^, ^18^, ^19^ ^]^ In multiple tissues, critical functional genes contain heterochromatin within NE segments adjacent to actively transcribed exons (Figure 1d). For example, H3K9me3 (a marker of constitutive heterochromatin) is deposited within TNNI3 (cardiac troponin) in human heart samples. This biallelically expressed gene must be transcribed throughout the human lifespan to maintain heart contractility (Figure S2, Supporting Information).^[^ ^20^, ^21^, ^22^, ^23^ ^]^

We wish to propose a radical hypothesis – described within this work – that nanoscale packing geometry is encoded in the positioning of exons, introns, and intergenic segments as projections of the functional layers of PDs observed on ChromSTEM tomography. This would pair NE segments with adjacent exons as a linear projection of the 3‐D volumes observed experimentally.^[^ ^9^, ^10^, ^12^, ^24^ ^]^ For this hypothesis to be valid, it requires 1) a length‐dependent pairing of NE segments with exons to generate volumetric ratios, and 2) that they be oriented in the direction of gene transcription. We demonstrate that both properties are observed. Even though we are unable at this time to prove this hypothesis mechanistically as it would require advances in ChromSTEM imaging to localize specific segments of the gene body—which is far beyond state‐of‐the‐art imaging capabilities—we provide correlative experimental evidence from nascent‐RNA sequencing, ChIP‐Seq, ATAC‐Seq, paired‐end tag sequencing (ChiaPET), and ChromSTEM tomography to support this hypothesis. While the proposed geometric system produces a mechanism to generate transcriptional efficiency, memory, and complexity, we show that it may come at increased mutation risk of the exposed branch points between domains. By analyzing oncogenes, we show mutation frequencies across all cancers correlating with genes containing branch point segments. Of particular interest is that this geometric system may have emerged in parallel with increased body plan complexity of metazoans, suggesting that packing geometry introduces a possible novel, non‐mutagenic system to increase information sampling based on physical properties. In sum, the proposed theory in this hypothesis generating study invites new avenues of research across diseases of aging, cancer, species evolution, and complex organ development rooted in physical genomics.

## 2 Results

### 2.1 Chromatin Domain Geometry Optimizes Transcription in the Human Nucleus

All methods encounter limitations in measuring chromatin at the smallest length scales due to the problem of missing information. Many tools provide sequence‐dependent chromatin interactions, including proximity capture methods (Hi‐C, Sprite, etc)^[^ ^25^, ^26^, ^27^ ^]^ and in situ hybridization (FISH) imaging.^[^ ^28^, ^29^, ^30^ ^]^ However, connectivity in a population may not equate to geometry in a single cell (Figure S2, Supporting Information). To complement connectivity measurements with nanoscale density, most studies employ super‐resolution imaging, accessibility assays, or variants of chromatin immunoprecipitation (ChIP‐Seq).^[^ ^10^, ^24^, ^31^, ^32^, ^33^, ^34^, ^35^, ^36^, ^37^, ^38^ ^]^ For example, it appears that antibody probe sizes used for super‐resolution imaging of chromatin cannot penetrate high density domains in situ. This results in high‐density and low‐density regions devoid of signal (Figure S3a–d, Supporting Information).^[^ ^39^, ^40^ ^]^ A separate problem is present for super‐resolution imaging using FISH probes that rely on formamide dehybridization. The denaturation process swells nanoscale domains, causing a loss of volume and density information.^[^ ^41^, ^42^, ^43^ ^]^ Due to these limitations of nanoscale imaging and molecular methods, there is limited knowledge of human gene geometry at the smallest length scales. While FISH has shown that a large chain or melt phenotypes are present for a few very genes, the observed distances are consistent with these genes being compressed in space (Experimental Section).^[^ ^44^ ^]^

It is worth considering the differences between ChromEM and conventional labeling on electron microscopy on the biological and physical implications of these structures. Due to the lack of specificity in staining on conventional electron microscopy, chromatin appeared as a near binary system of high and low density. On ChromSTEM imaging, density within PD volumes is a continuous density gradient. As a result, instead of chromatin being binarized into competing structures, each volume had a distribution of contents based on its size and packing efficiency. Larger structures with high efficiency were enriched in high density, presumably heterochromatin centers that gradually decreased toward a periphery comprised of presumably euchromatin. Smaller volumes have similar organization, but the total content shifts towards a smaller core. This underlying organization was indeed supported by multi‐color super resolution imaging, showing that heterochromatin (H3K9me3) was positioned such that RNA polymerase II and euchromatin markers were positioned around a “core”.^[^ ^8^ ^]^ In this context, it is necessary to utilize a polymer framework to integrate physical observations with molecular behavior. The fractal dimension provides a useful physical framework to measure and quantitatively describe how the chromatin polymer is packed into the PD volumes. PD geometry can be described as a mass fractal as follows: for large genes and loops, the genomic length (*N*) of a gene/loop (e.g., in nucleosomes) fills space as a function of radius by the dimension, *D*. This is analytically described by the equation, *N*∝*r^D^*; a property confirmed on ChromSTEM tomography.^[^ ^9^, ^10^, ^13^, ^45^ ^]^

In many ways, it is conceptually easier to consider two limiting cases of mass fractals to understand the structure of nanoscopic packing domains. A random walk without attractive potentials or confinement has scaling *D* = 2, which has a very loose distribution within a volume (Figure S4a, Supporting Information). An extension of this is a confined random walk, the fractal globule, which will have a scaling of *D = 3* but has a uniform distribution of density (Figure S4b, Supporting Information).^[^ ^45^ ^]^ It may be tempting to assign chromatin as an open state to *D = 2* and closed state to *D* = 3 based on prior experimental observations.^[^ ^45^ ^]^ However, this is not observed experimentally on ChromSTEM as discussed above. Instead, chromatin throughout the nucleus organizes into domains with *D* values ranging between 2.2 and 2.8 with a radius ranging between 25 and175 nm. This indicates non‐uniform density: a gradient is present that radially decays from a high‐density interior to a low‐density periphery where the fractal dimension describes the statistics of the chromatin chain filling the space.^[^ ^9^, ^10^ ^]^ Unlike condensates with uniform density, mass fractal volumes with a high fractal dimension have both a higher packing density as well as an increase in the effective surface area for chemical reactions to occur.^[^ ^13^ ^]^ Collectively, the result is that packing at the nanoscale couples high density (heterochromatin cores) with a lower density outer zone (euchromatin) instead of alternating volumes. The intermediate density between these two regions appears to be an ideal zone for transcriptional reactions to occur. Based on these zones, it was shown that disruption of heterochromatin enzymes can experimentally decrease RNA synthesis due to the disruption of the ideal zone supported by the core element. Furthermore, it can self‐assemble through transcription‐mediated loops, first generating nascent (small, poorly packed) domains. While these results indicate that transcription appears to happen at the intermediate region, it remains to be understood where the geometric information for such a system is stored.

**Figure 2:**
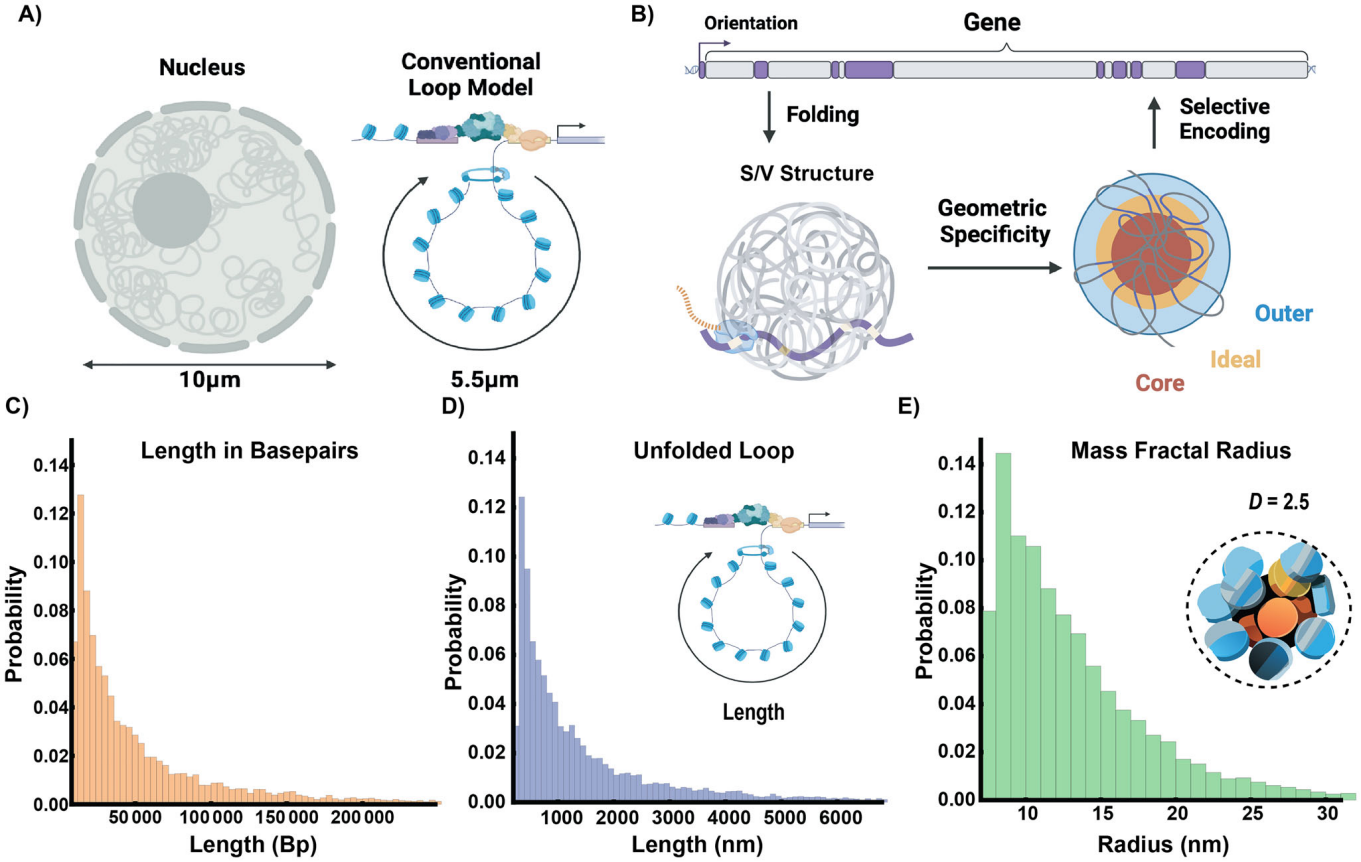
Packing domains as a geometric solution to optimize nuclear volume and transcriptional efficiency. A) Loops have a broad range of sizes with many larger loops (>100 Kbp) generating lengths that would span the human nucleus without a system for efficient packing. B) Proposed framework that the position of exons, introns, and intergenic elements produces a system to reliably generate reaction volumes. Exons with short intronic sequences fold into an ideal zone within a volume generated by NE DNA (the ideal zone as a surface‐area to volume – SA/V – of the total volume). The resulting volumes represent the structures observed on ChromSTEM imaging. The continued selection for elements across broad‐timescales results in an encoding within the genome. C‐E) Transformation from beads on a string into mass‐fractal volumes compresses genes from micron‐length chains into nanoscopic volumes.

**Figure 3:**
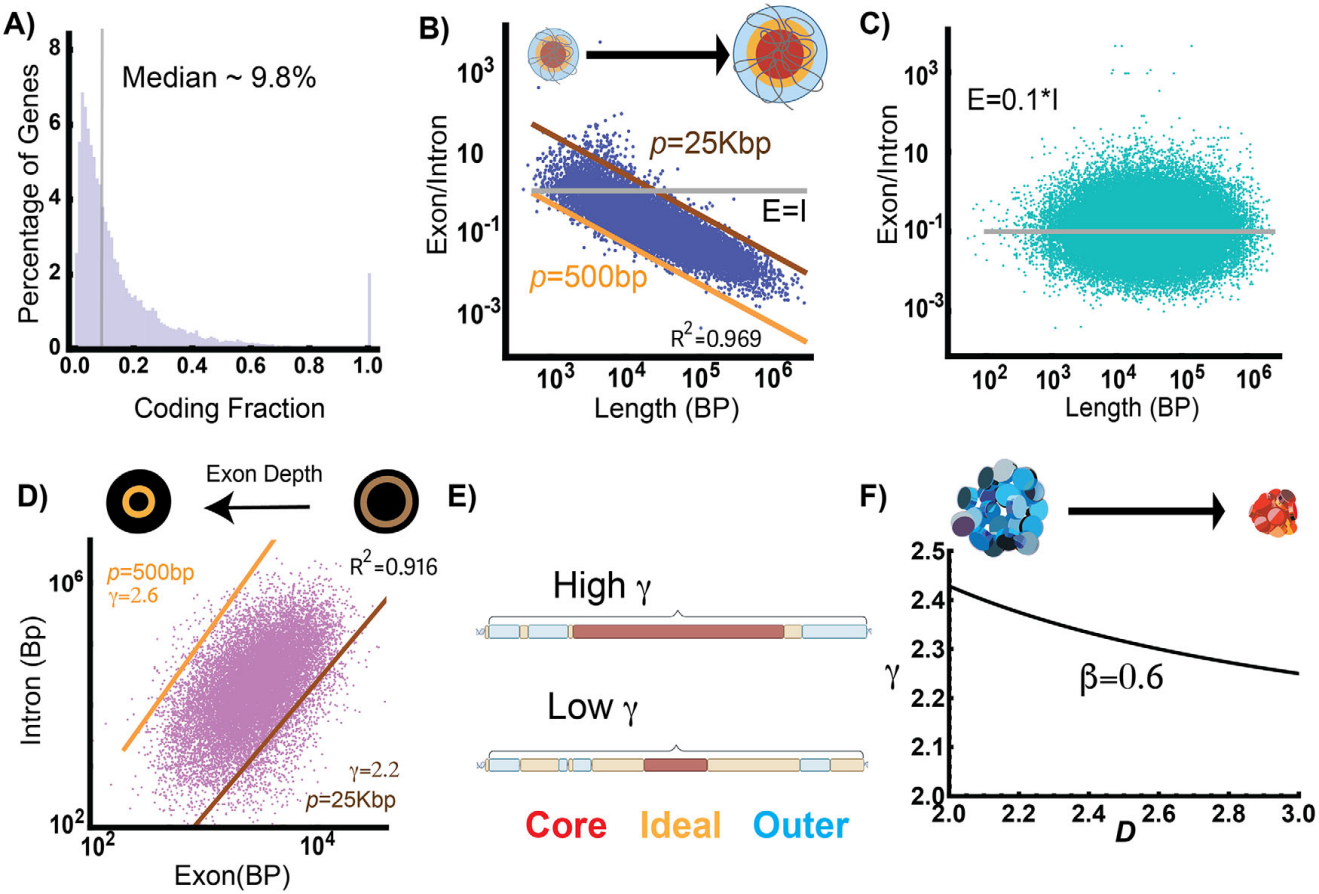
Introns and genes are geometrically linked to exon length by physical principles. A) Histogram of protein‐coding genes in the human genome showing that the median fraction is ≈9.8% exonic with a subset of genes that are almost completely exon. B) Plot of the ratio of exon (ideal zone)/intron (total volume) compared to the gene length of human genes. The constant, *p*, reflects the position along a domain volume. Here, *n is* set to 1. As length increases, as expected, the volume ratio decreases as expected for a distribution of reaction volumes. C) Randomization of exon/intron segments results in the statistically grounded null hypothesis of no relationship between length and exon/intron ratios, with a fraction approaching the median of 0.1. D) Intron length is a power‐law of exon length for protein coding genes with values of *p* and γ as reported. E) Schematic representation of gene composition in relation to *p* and γ, indicating that high γ indicates more non‐exonic volumetric elements are present within a segment. F) Relationship between γ and *D* depends on the proportion of the exons making up the ideal zone where β = 1 indicates the entire exon contents are confined to a hard surface.

A hypothesis we propose and describe is that large genes and loops are partially constrained (packed) into domain volumes. We can consider muscle and myosin heavy chain 1 (Myh1) as a representative example for comparison between models. Myh1 is ≈26,000 basepairs, which translates into ≈130 nucleosomes. A nucleosome is approximately a cylinder composed of ≈200 bp with a diameter of ≈11 nm and height of ≈5.5 nm. Stacking end‐to‐end to produce a fully stretched state produces a ≈715 nm loop. For scale, chromosome 2 is composed of 243Mbp, contains ≈1200 genes, and in mature muscle cells has a radius of ≈1.5 µm (Figure S5, Supporting Information).^[^ ^46^ ^]^ Since muscle function requires the synthesis of hundreds of genes, many of which are quite large, accounting for space is crucial. Outside of muscle, a similar problem arises with loops. Many loops are over 100 kbp (which translates into 5.5um), a length which would span the radius of the nucleus if not folded in space (Figure 3a, Figure S6, Supporting Information).^[^ ^47^, ^48^ ^]^ The hypothesis proposed is that packing information is stored by exons non‐randomly pairing with NE in order to generate the functional layers observed on ChromSTEM imaging (**Figure** **2**b).

### 2.2 Human Genes Are a Predictable, Power‐Law Geometric Assembly of Exons and Introns

As a consistent convention across the different types of RNA products, we refer to DNA transcribed and processed into functional RNA as “exons” independent of the RNA product class (mRNA, lncRNA, pseudogenes, etc). Similarly, we refer to infrequently transcribed (NE) DNA as introns (within a gene body) and intergenic segments (between bodies).^[^ ^14^, ^49^, ^50^ ^]^ We recognize that non‐exonic DNA has many sequence‐specific regulatory functions and that the RNA products also have unique functions.^[^ ^51^, ^52^, ^53^ ^]^ As the focus is on packing geometry encoding novel information, we intentionally do not make sequence specific measurements in the derivation nor restrict the analysis of the genome to avoid regions based on sequence content. Instead, this investigation focuses on the hypothesis of whether 3D geometry, guided by transcription, could project exonic and non‐exonic elements in a manner that can generate the reaction volumes within the human genome consistent with the observations above. Specifically, we examine if exonic and non‐exonic elements are coupled such that volumes could arise where transcriptional reactions are most efficient, the ideal zone, supported by the packing volume. This geometric theory is as follows.

Based on the continuous density gradient of PDs, positioning exonic elements to the ideal region of the volume would depend on an inverse relationship of the ratio of exons‐to‐introns as a function of gene length. This is because the length of a segment (gene/loop) folded into a domain volume translates into the radius occupied as a function of *D* (Figure 2d–f).^[^ ^9^, ^10^, ^38^, ^39^, ^54^, ^55^, ^56^ ^]^ In effect, the ideal zone volume would represent a “surface area” (a shell) of the total PD volume. The inverse relationship of the ideal reaction zone to the total domain volume mirrors that of the surface‐area‐to‐volume ratio observed in spheres (Figure S7, Supporting Information), which decreases as a function of the radius (Figure 2b, Experimental Section). Here, it refers to content organized into zones within a total volume: a shell/volume (S/V) relationship. For this to occur, genes are hypothesized to contain enough NE DNA to fold into domains to position exons for efficient transcription. An alternative to the domain hypothesis would be that genes assemble into beads‐on‐a‐string without a higher‐order assembly. In sharp contrast to this inverse relationship of domain volumes, beads‐on‐a‐string results in a linear relationship between the exonic fraction to the NE length (Figure S7, Supporting Information). In a chain assembly, as the number of nucleosomes increases, the amount of accessible DNA increases proportionally to length (Figure S7, Supporting Information). Finally, the null hypothesis is that no volume‐producing geometric relationships exist. In the null hypothesis, exonic and non‐exonic elements within the genome are randomly spaced without any constraints. In the null hypothesis configuration, gene content would be a random assortment of exonic and non‐exonic contents without any restrictions on the positioning of intronic DNA as a function of length. In the null hypothesis, one would reasonably observe that exon length is ≈10% of any gene body independent of the length. This is because only 10% of the gene length on average is exonic. Instead of random assemblies of exonic and non‐exonic contents, the human genome contains a broad distribution in the exon fraction within genes (**Figure** **3**a, interquartile range of 4.6–21.1%) that is length dependent, as we describe below.

Using the publicly available human reference genome GRCh38, with annotations from RefSeq, we tested whether the inverse relationship between contents occurs across the human genome consistent with the domain‐forming hypothesis.^[^ ^57^ ^]^ We calculated the ratio of exons to introns versus total length for all protein‐coding genes, finding that most human genes are described by the geometric principle consistent with volumetric packing observed on ChromSTEM imaging (Figure 3b, Experimental Section). This relationship is defined as follows by Equation (1) where *p* is a scalar in basepairs:

(1) $$\frac{\mathrm{Exon}}{\mathrm{Intron}\;}≅\frac{p}{\mathrm{Lengt}h^{n}}$$

We observe most genes are well described by a *p* range from 500 to 25 000 bp, which is consistent with ≈2 to 125 nucleosomes when *n* is close to 1 (*R* ^2^ = 0.967, p‐value < 0.001 compared to randomized controls). This is observed both for individual gene isoforms or all the variant isoforms within the RefSeq database (Figure S8, Supporting Information). An alternative explanation for such a trend is that as gene length increases, the exonic fraction decreases. If this were the case, this pattern would be observed even if exons were randomly redistributed. However, random repositioning of exons produces a length‐independent constant ratio of E/I of ≈0.1 that is instead consistent with behavior of the null hypothesis. These findings suggest that gene length is non‐randomly associated with exon content (Figure 3c). We next account for the possibility that the observed ratio decreases due to length alone, therefore we tested if intron length is geometrically coupled to exon length as a power‐law consistent with the formation of domain volumes.

To be consistent with the domain forming hypothesis, one should expect to observe intron content scale as a power law with a value between 2 and 3 of the exons contained within the gene. While this would not prove causation of function, this correlative finding would support the hypothesis that compositions of genes are related to packing geometry observed on ChromSTEM imaging. Supporting the proposed volumetric hypothesis, the scaling relationship is indeed observed (Figure 3d). This is quantitatively described by Equation (2) where *p* ranges from 500 to 25 000bp:

(2) $$\mathrm{Intron}\propto \left(\mathrm{Exo}n^{\gamma }\right)/p$$

Understanding the function of the scalar, *p*, and the exponent, γ, involves considering how a polymer, such as chromatin, translates from linear information into 3‐D packing volumes. Along the linear genome, *p* and γ likely reflect the exonic information contained within a segment as it is folded into a functional volume. A chromatin chain segment with a lower γ (≈2) produces less linear spacing than larger γ (≈3) in order to fold into a functional 3D reaction volumes. The value of *p* indicates where along the depth of a volume a segment is capable of being positioned to interact with enzymes such as RNA polymerase. From this perspective, a smaller *p* value suggesting a segment resides deeper within the generated volume, and a larger value the inverse.

Since cells within the human body have a broad distribution of sizes and packing states, the central hypothesis suggests both degrees of freedom interact to accommodate an effective packing configuration across multiple conditions. This potentially encodes a system that is sensitive to factors including the concentration of nucleosome remodeling enzymes, ion concentrations, nuclear size, existing domains, etc, that are difficult to measure in every human cell. Generally, a high *p* and low γ state produces high information density in the linear chain. As a result, structural genes such as Myh1 (*p* ≈7800, γ = 2.2) are proposed to adopt more complex geometries with some degree of packing present. It is worth noting that in the proposed hypothesis, γ is inversely proportional to *D* as the transformation of the chain into the occupied volume, quantified by:

(3) $$\gamma =1+\frac{D}{D-\beta }$$

where β defines the fractional dimension of exonic contents on the ideal zone (Figure 3e,f, Experimental Section). A reasonable biological interpretation of these parameters is as follows. As the fractal dimension describes how the genome fills 3‐D space, γ provides the reciprocal information on spacing in the chain to effectively produce a configuration such that the genomic contents are positioned within this volume. As the ideal zone reflects the S/V of the total structure, β, captures how the chain fills the ideal zone shell within the total domain volume.

The values of these scaling exponents are physically indicative of whether exonic and NE regions are capable of producing the 3D volume relationships of PDs. Considering the limiting case where 3D packing geometry is not observed (e.g., a chain assembly), it results in a β = 0 producing the following parameters γ  =  1, *c*  =  1, and *n*  =  0 with *I*∝*E* and *E*∝*L*. Effectively, in this configuration, the entire gene segment is a beads‐on‐a‐string chain. On the other hand, a strong packing geometry corresponds to 2 ≤ γ ≤ 3, *c* < 1, and *n*  ≈  1. Our observations within the experimental data from the human genome are consistent with the latter case of packing geometry. Although these descriptions are not mechanistically proved due to limitations in imaging technologies to pair individual genes with 3D volumes on ChromSTEM, as we describe in detail within this manuscript, multiple lines of evidence support the central hypothesis of the genome encoding packing information. Finally, since we propose that these properties are related to the activity of transcription, we verified that these scaling behavior arise in independent of the final transcript type (pseudogenes, lncRNA, etc) by analyzing these scaling behaviors in non‐protein coding RNA gene classes (Figure S8, Supporting Information).

### 2.3 Gene Positions on Human Chromosomes Reflect the Projection of 3D Domain Volumes

**Figure 4:**
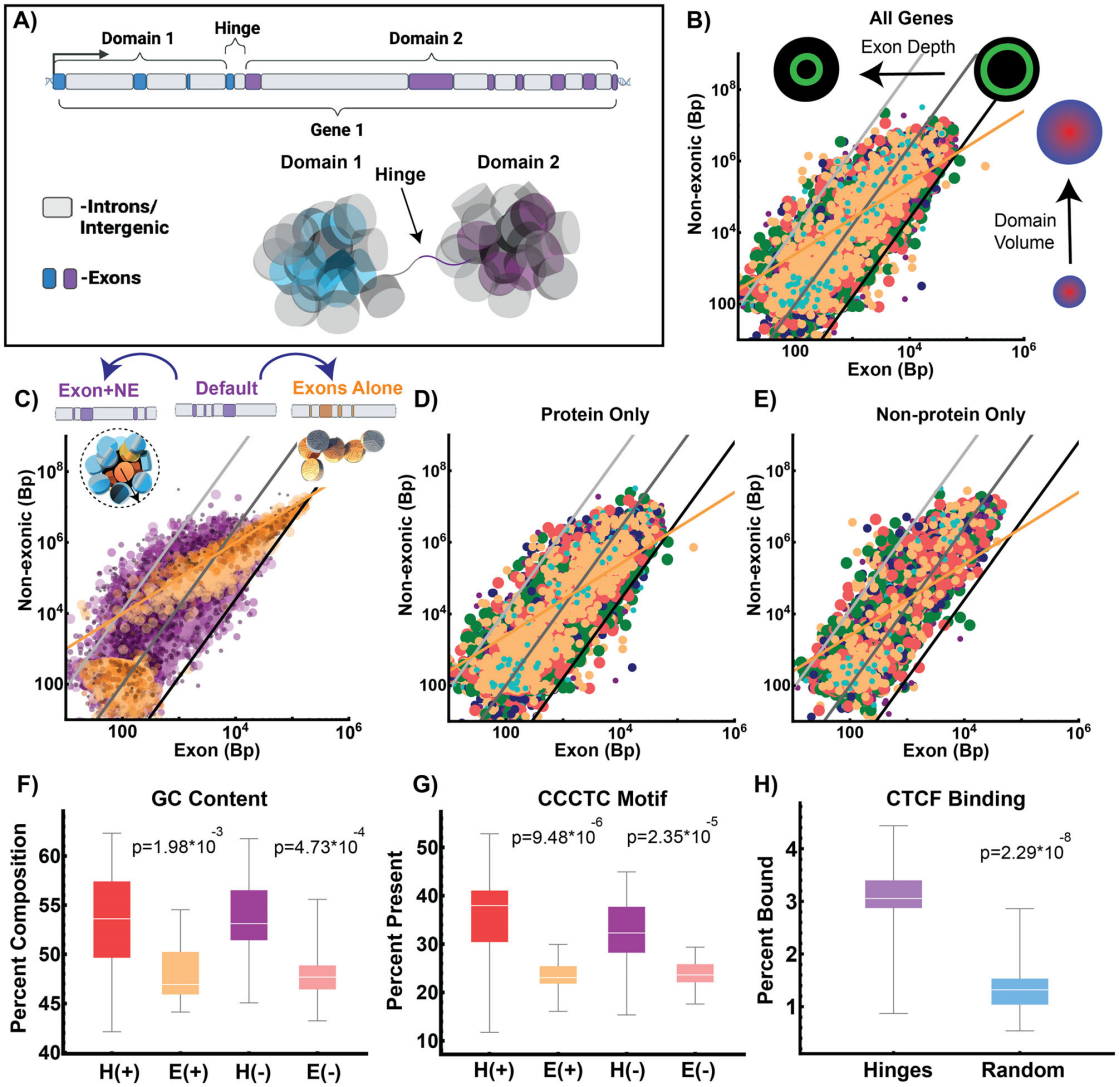
Exons are non‐randomly coupled to adjacent volumetric DNA to generate power‐law segments independent of the final RNA product. A) Schematic representation of the organization of a gene on a chromosome segmented into two separate domains generated by a hinge element. The reaction volumes are produced by the volumetric DNA to guide the position of exons to ideal reaction zones. B) Analysis of the structure of human chromosomes in the positive strand orientation showing power‐law assemblies of nontranscribed (volumetric) elements scaling as a power‐law of exonic (ideal zone) elements. C) Randomly redistributing an exon with adjacent volumetric DNA conserves power‐law distribution. In contrast, randomly distributing exons results in a linear distribution of segments. D,E) Comparison of organization generated by considering only (D) protein coding genes compared to (E) only non‐protein coding genes in the positive strand orientation. In both cases, chromosomes assemble into power‐law segments. F) Sequence composition analysis of “hinges” demonstrates enrichment of GC content compared to exons. G) CCCT‐C motif analysis demonstrates enrichment of possible CTCF‐binding sites within “hinge” elements in either strand orientation. H) Observed frequency of CTCF‐binding on hinge positions in HCT‐116 cells demonstrates enrichment compared to random positions of similar size throughout the genome.

We next investigated if these principles generalize to the positioning of exons, introns, and intergenic segments on whole chromosomes. In this analysis, we investigate all somatic chromosomes (1 through 22) as well as the X‐ and Y‐chromosomes. We do not omit any specific genomic segments, chromosomes, or regions based on sequence‐based features such as sequence contents (AT/GC) or gene density. Experimentally, it was observed that transcription appears to guide domain formation, which suggests that genes oriented in *cis* may share domain volumes within the chromosome. In this hypothesis, intergenic segments could have similar volumetric properties as introns to support domain formation, i.e., both introns and intergenic segments behave as volumetric DNA elements. On both ChromSTEM imaging and in polymer simulations, packing domains are separated by an interdomain space formed by DNA.^[^ ^9^, ^10^, ^38^, ^54^, ^58^, ^59^ ^]^ This finding indicates that domains are not a series of overlapping volumes but have some small spacing that separates them by ≈10–50 nm. From these theoretical and experimental observations, we hypothesized that this linker segment contains an element that is recognized by Pol II (for domain generation) and contains enough non‐exonic DNA to generate a stable separation between the two domains. We refer to this as a “hinge” element. Hinges are composed of an exon+NE segment that is small enough not to become packed as independent domains but long enough to produce spacing between the domain volumes. We describe it as a “hinge” in that we hypothesize its activity depends on whether it is actively engaged by active Pol II (being transcribed) to form two separate segments. These two segments would then have the capacity to fold into two domain volumes by the proposed theory. Reciprocally, a domain segment will be formed by the span between the two hinges that are actively engaged. When not engaged by Pol II, a hinge segment may revert (fold back into a volume), resulting in an alternate geometric configuration (**Figure** **4**a). An alternative possibility is that hinges are only composed of transcribed segments (exons), but we hypothesized that the supercoiling generated by polymerase could potentially alter the composition of the domains continuously (domains could merge or separate continuously).^[^ ^60^, ^61^ ^]^ Finally, we consider the case where hinges were completely non‐exonic, but these would lack a mechanism for Pol II to guide domain segmentation that is observed experimentally. Since hinges are not domains, they are likely no more than a few nucleosomes long (<1000 bp, ≈50 nm long). Above this range, they would potentially interact with nucleosome remodelers via the mechanisms that produce domain volumes, limiting their ability to achieve spacing. For the purposes of this manuscript, we focus on an example case where hinges are defined as being an Exon+NE segment less than or equal to 300 bp (1–300 bp, 1–2 nucleosomes).

By accounting the presence of hinges composed of exon+NE to separate domains in space, we find that every human chromosome (somatic as well as X‐ and Y‐) results in assemblies that are consistent with into power‐law packing ratios of domains (Figure 4b, Figures S9 and S10, Supporting Information**;** R^2^ 0.965–0.970, p‐value 3.27 × 10^−4^–4.66 × 10^−6^ for the negative and positive orientations, respectively). This organization resembles individual genes described by Equation (3), with *p* and  γ adopting the same meaning:

(4) $$\mathrm{NE}\propto \left(\mathrm{Exo}n^{\gamma }\right)/p$$

This finding suggests that exons are non‐randomly linked with their adjacent NE neighbor to produce reaction volumes. Since this is difficult to test experimentally, we test the hypothesis analytically by randomizing the positions of exons alone and comparing to randomizing exon+NE pairs (Figure 4c). We then compared the packing ratios produced when 1) we randomly repositioned the exon positions on their respective chromosome compared to 2) randomly repositioning exons with the adjacent NE (exon+NE). In this approach, the total exonic and NE content remains nearly constant (less 5% differences) on each chromosome, but the elements are redistributed. If one were to observe that PD volume distributions can be present from just exon position alone, it would suggest against the central hypothesis. Conversely, if the repositioning of exon+NE together preserves the statistics of packing segments, it indicates a non‐random coupling within the genome. When exons are distributed alone, the power‐law compositions are lost and the produced segments instead resemble linear beads‐on‐a‐string ratios (Figure 4c,d; R^2^ 0.89–0.97, p‐value 3.37 × 10^−4^–4.29 × 10^−6^ for the negative and positive orientations, respectively). In contrast, when exon+NE are randomly distributed together, the power‐law patterns are maintained. This supports the hypothesis that exonic segments are non‐randomly paired to an adjacent NE segment in 3‐D volumes. If this is intrinsically driven by transcription itself and not the final RNA product, these patterns would be present in either protein coding or non‐protein‐coding segmentation. Independent of the type of RNA generated (protein coding RNA, non‐protein coding RNA), chromosomes assemble into power‐law segments of exonic‐/NE‐ elements (Figure 4c,d).

Collectively, these findings are consistent with the central hypothesis that exons, introns, and intergenic segments are geometrically coupled in transcriptionally mediated reaction volumes. We next characterized the sequence properties of the described “hinge” elements that were derived to act as physical spacing elements between PDs. We first analyzed the GC content within hinge regions, finding that these regions were unexpectedly enriched in G/C content (≈53% for hinges 1–300 bp, p‐value between 1.9 × 10^−4^ to 4.73 × 10^−5^ for the positive and negative oriented hinges, respectively; Figure 4f). Owing to this finding and the proposed theory that hinges act as spacing elements guided by transcription, we hypothesized that these regions would be enriched for core CTCF binding motifs (CCCTC). To test this hypothesis, we measured the relative frequency of hinge regions containing CTCF motifs compared to exons as a control. Consistent with the hypothesis that hinges act as possible spacing elements between segments, we observe significant enrichment of CTCF motifs (37% versus 22% for exons, p‐value 9.48 × 10^−6^ in the positive orientation and 32% versus 24% with p‐value of 2.35 × 10^−5^ in the minus orientation; Figure 4g). Next, we measured the rate of CTCF binding to hinge regions, observing that 3% of hinges were bound in HCT‐116 cells compared to under 1% for comparably size random positions (p‐value 2.92 × 10^−8^, Figure 4h). Although hinge elements were derived based on ChromSTEM observations, these sequence specific findings raised interesting questions about the role of hinges as elements that can define physical segments guided by transcriptional reactions.

### 2.4 Predictions of Genome Geometry Are Observed Across Genomic Methods

The central hypothesis proposed in this manuscript – exons, introns, and intergenic segments are non‐randomly coupled by the 3‐D volume geometry – introduces five dependent and testable hypotheses, even as testing the central hypothesis directly is challenging:

H1) The DNA content of predicted segments agrees with experimental observations on ChromSTEM,

H2) Exons have a higher likelihood of interacting with Pol II based on their position,

H3) RNA synthesis decreases from the introns of long genes from suboptimal positioning,

H4) Hinge segments are enriched for features of active transcription, and

H5) Accessibility within segments should resemble a power‐law packing ratio shifted to the outer zone.

Although these hypotheses cannot definitively prove the novel positioning hypothesis mechanistically, as it would require manipulation and synthesis of genetic elements that are paired with ChromSTEM imaging, the proposed central hypothesis requires all of them to be sustained. Therefore, we tested whether these dependent hypotheses are supported by experimental data. The following are experimental tests of these dependent hypotheses.

### 2.5 Dependent Hypothesis 1

**Figure 5:**
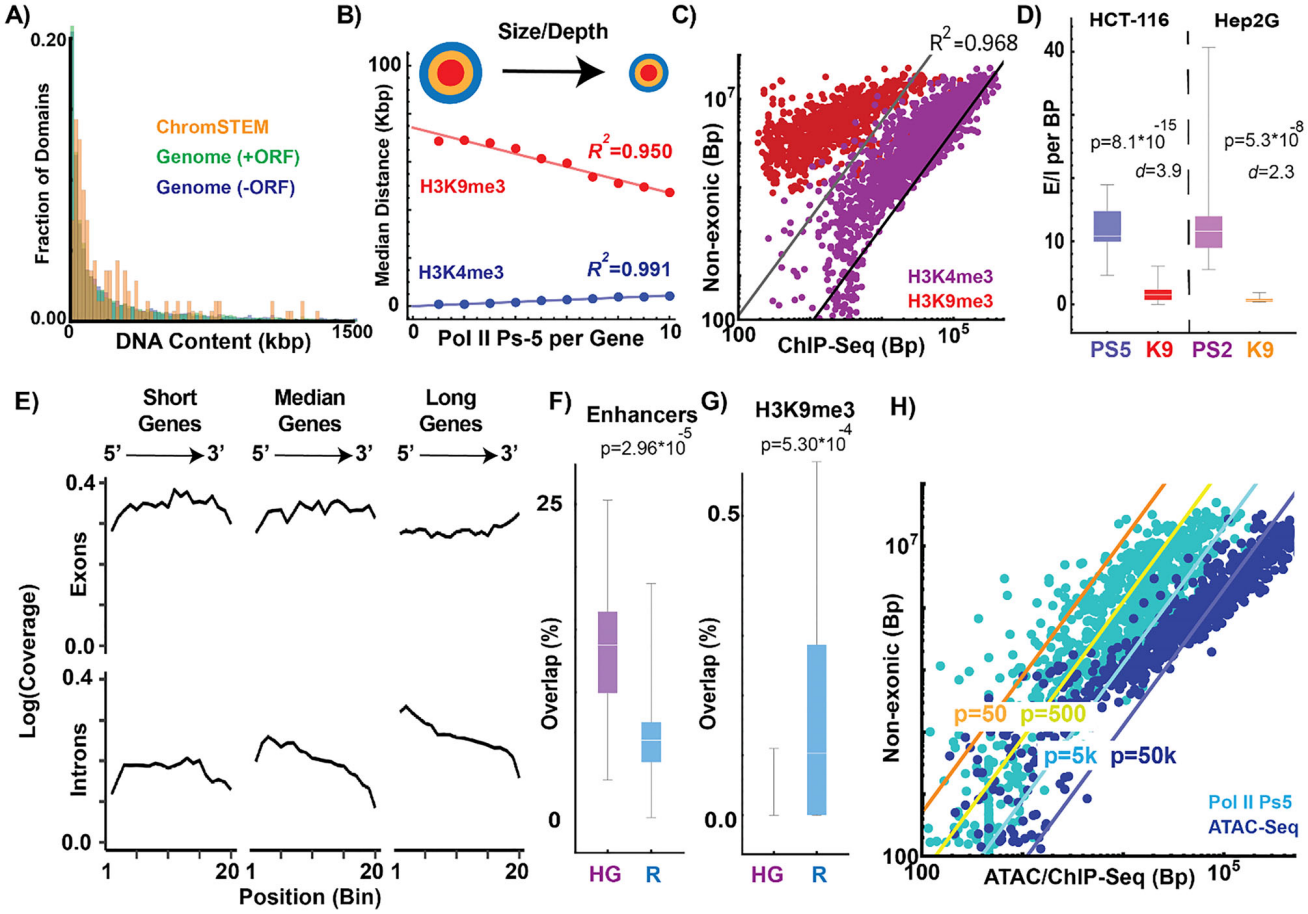
Exons, introns, and intergenic segments are non‐randomly positioned into reaction volumes. A) Comparison of the DNA content observed in ChromSTEM packing domains compared to analytical predictions of the model when hinge length is less than 300 bp demonstrating similar distributions in content. B) Analysis of distance between active Pol II (PS‐5) to the nearest core element (H3K9me3) as a function of Pol II Ps‐5 density. Shallower depths and smaller domains would result in a higher concentration of Pol II Ps‐5 per segment and would be expected to have a shorter distance to a core as is experimentally observed. C) Analysis of the ChIP‐Seq content of heterochromatin (H3K9me3) and promoter associated euchromatin (H3K4me3) within theorized volumes from the model in HCT‐116. Consistent with underlying theory, chromatin segments are composed of both elements with positioning H3K4me3 coinciding with position of exon elements while heterochromatin is positioned to deeper layers. This partitioning suggests that heterochromatin is coupled with euchromatin in genetic segments. D) ChIP‐Seq analysis of active isoforms Pol II Ps‐5 in HCT‐116 cells and Pol II Ps‐2 in Hep2G cells within gene bodies (exons and introns) demonstrating preferential localization of polymerase onto exons per basepair length. If polymerases were uniformly distributed throughout a gene body equivalent coverage per basepair would be observed. As a control for read‐coverage bias, this was compared to H3K9me3 (K9), which preferentially localizes to introns. E) Nascent RNA‐seq analysis in HCT‐116 demonstrates nearly uniform synthesis of short introns and exonic sequences independent of gene lengths. In contrast, longer genes demonstrate decreasing synthesis of RNA in the direction of the reading frame consistent with Pol II having ideal reaction positions. F) Analysis of hinge segments in HCT‐116 demonstrates an enrichment toward transcriptionally active features and enhancer positions compared to randomly generated segments (R). G) In contrast, a very small percentage (<1%) of hinge positions overlaps with constitutive heterochromatin. H) Analysis of active Pol II (Pol II Ps5) in activated power‐law segments compared to the associated accessibility (ATAC‐Seq). Pol II Ps5 primarily localizes in regions that are consistent with exon positioning in the theorized model. Notably, accessibility appears to reside in a further distal zone.

To test H1, we utilized existing ChromSTEM imaging data from HCT‐116 cells. The DNA content observed on ChromSTEM domains can be calculated by converting intensity into content using the mass‐fractal relationship.^[^ ^9^, ^16^ ^]^ Although we cannot identify the sequence composition on ChromSTEM, we can compare the distribution of sizes between theory and experimental measurements of PDs. Consistent with theory predictions, the DNA totals generated by the segmentation described are very similar to those experimentally observed (**Figure** **5**a, Mann–Whitney U‐Test 0.89 for negative reading frame and 0.93 for positive reading frame) but not for segments generated from randomly positioned exons (Figure S11, Supporting Information, Mann–Whitney U‐Test <10^−45^).

### 2.6 Dependent Hypothesis 2

H2 depends on three observations. Due to the limitations of some feature mapping to the Y chromosome on ‐omics methods, we compared chromosomes 1–22 and X‐ to the observations. First, one would observe a length‐dependent coupling between functional zones (cores, active Pol II, and euchromatin) that could reflect domain volumes. Specifically, the higher volumetric ratio in small domains produces a higher concentration of Pol II with a shorter linear distance to a core (H3K9me3). In contrast to packing features, chromatin organized as a chain assembly would display an increasing distance between Pol II to H3K9me3 as these would represent opposing functional states. Using ChIP‐Seq data available through ENCODE,^[^ ^17^, ^18^, ^19^ ^]^ we tested if the coupling between transcription and heterochromatin is consistent with domain volumes by measuring the distance between active Pol II (Ps2/Ps5) and the nearest H3K9me3 as a function of the polymerase concentration.^[^ ^12^ ^]^ A complete list of the ENCODE datasets used with the number of replicates, is included in SI Table 3. We performed this analysis on induced pluripotent stem cells (GM23338), HepG2 hepatocellular carcinoma cells, and SK‐N‐SH neuroblastoma cells using data from ENCODE. If Pol II is guided by geometric position, an inverse distance relationship is likely to be conserved across models (higher Pol II concentration resulting in shorter distances). Further, because iPSCs are highly enriched in euchromatin,^[^ ^62^, ^63^ ^]^ the theory predicts them to have the smallest domains resulting in both the highest volumetric ratios and shortest distances. These predictions are observed: all three cell lines have the inverse distance relationship and iPSCs have the shortest distances on average (Figure 5b, Figure S12, Supporting Information). Second, H2 indicates that exons can be positioned to geometrically interact with polymerase, euchromatin, and heterochromatin based on the volumetric structure. Specifically, one would expect markers such as H3K4me3 and active RNA polymerase II to localize in the projection of the hypothesized ideal zone mainly composed of exons whereas, conversely within the same segment, H3K9me3 would localize to deeper layers. These observations within the segments would be observed as a shift in the *p* required to position these elements such that H3K4me3 and Pol II would range between ≈250 and 25 000 bp (similar to the contents in the exonic volume layer) and H3K9me3 to values below this range (1–50 bp) to deeper layers. These behaviors are indeed observed experimentally in HCT‐116 cells (Figure 5c).

### 2.7 Dependent Hypothesis 3

For H3 to hold, there must be some evidence of actively transcribing Pol II to preferentially binding to exons when accounting for the differences in segment length. In support of H3, active Pol‐II is nearly ten times more likely to found to an exon than an intron in both Hep2G and HCT‐116 cells when accounting for length (Figure 5d and p‐value <10^−3^). For H3 to hold, one would observe a 5′ versus 3′ bias in the amount of RNA produced within long gene segments. In bulk RNA‐seq, it has been observed that a “saw‐tooth pattern” occurs, potentially as the result of the delayed processing of RNA by the splicing enzymes or the decay in introns.^[^ ^64^ ^]^ Although nascent RNA‐seq (EU‐Seq) cannot exclude additional considerations such as differential rates of co‐transcriptional splicing, it provides a method to estimate the relative rate of mRNA production based on gene size and the positioning of segments. We tested whether a length‐dependent rate of synthesis was observed in exons and introns as a function of the traversed length. Consistent with H3, there is a length‐dependent decrease in RNA synthesis in 5′ to 3′ orientation for introns of long genes (Figure 5e) that is not observed in the synthesis of exons or short intronic segments. To verify this observation, we performed RNA immunoprecipitation sequencing (RIP‐Seq) for RNA bound to actively transcribing Pol‐II Ps2 in HCT‐116 cells. Consistent with nascent RNA sequencing and ChIP‐Seq findings, we observed preferential RNA bound to Pol‐II Ps2 on exons and the flanking segments of short introns (Figure S14, Supporting Information). This behavior was observed even within individual genes, with long intron segments appearing to have decreased RNA synthesis throughout the body even as adjacent short introns were transcribed (Figure S14, Supporting Information). Further, we observe a length dependent decrease in RNA binding within long introns in the 5′ to 3′ orientation. Although additional investigation into splicing regulation is required, these results suggest preferential efficacy of RNA polymerase II within the exons of long genes.

### 2.8 Dependent Hypothesis 4

We used a similar approach to investigate H4, testing if hinge segments have preferential patterns in enrichment for active transcription. We analyzed data from ENCODE and the Atlas of Enhancers^[^ ^2^ ^]^ in HCT‐116 cells for hinges positioned in the human genome (HG) versus hinges generated by randomly repositioning exons (R). Hinge positions behaved as hypothesized, with enrichment in euchromatin marks and enhancers and depletion of heterochromatin markers compared to the random genome. In the human genome, ≈6.7% (interquartile range 6.1–7.5%) were bound by active RNA polymerase II (Pol II‐Ps5), 10.6% (interquartile range 8.6–11.6%) were marked by H3K27ac, 9.4% (interquartile range 8.6–9.8%) were marked by H3K4me3, and 35.2% of annotated enhancers contained at least one hinge element (interquartile range 28.3–40.2%) (Figure 5e,f, Figure S13, Supporting Information). In contrast, hinge positions were depleted of heterochromatin modifications with H3K9me3 occurring less than 0.11% of the time (interquartile range 0.0–0.11%) and H3K27me3 occurring 1.2% of the time (interquartile range of 0.9–1.8%, Figure 5g,h, Figure S13, Supporting Information). Collectively, this indicates, as proposed, that the act of transcription on hinge segments may facilitate the generation of domain geometry in a manner consistent with the proposed central hypothesis.

### 2.9 Dependent Hypothesis 5

H5 depends on the accessibility of chromatin within a packed volume compared to a beads‐on‐a‐string chain. There have been several molecular mapping technologies to resolve nucleosome positioning such as DNAse‐Seq, ATAC‐Seq, and Fiber‐Seq, among many others. These techniques rely on DNA modifying enzymes interacting with a chromatin segment as a function of accessibility. With respect to a 3D volume observed in PDs, one would expect a similar S/V pattern to exons that is shifted to shallower depths within the volume. This occurs because the continuous density gradient transitions from the ideal zone (enriched in active polymerases, H2 and H3) decreases further toward the periphery. As a result, one would observe that the packing ratio (accessibility/NE) within each segment would have similar scaling but with a larger *p*. Utilizing ENCODE data of transcriptionally active Pol2‐Ps2 and ATAC‐Seq, we measured the packing ratio in comparison to RNA polymerase coverage in the predicted segments, observing that these patterns indeed occur. Pol‐II Ps5 was observed to occupy as expected the regions enriched for exons with ATAC‐Seq accessibility extending to the periphery (Figure 5h).

In sum, the dependent hypotheses are supported by correlative experimental evidence across independent genomic modalities. While individually they can be explained by alternative mechanisms, the novel hypothesis that exons are non‐randomly coupled to NE to generate domain volumes, in our view, best explains the sum of the evidence.

### 2.10 Genome Geometry Suggests Transcriptional Loops Are Efficiently Packed

Having demonstrated that the volumetric pattern of domains could be projected onto the positioning of exons, introns, and intergenic segments, we then studied whether they experimentally intersect with gene transcription. Based on the proposed theory, this created a hypothesis that transcriptionally active loops (mediated by Pol II) are packed in a manner that resembles packing domain volumes.^[^ ^65^ ^]^ This is because durable volumes (packing) could produce high‐frequency loops due to spatial confinement. To test this hypothesis, we used publicly available data through ENCODE to analyze the composition of Pol II loops defined by ChiaPET.^[^ ^65^ ^]^ We partitioned loops into very strong loops (>20 events) and compared them to more transient loops (<5 loop events). We then analyzed the genetic composition of these two groups. Consistent with the hypothesis that loops are packed similarly to domain volumes, very strong loops contain exon/NE ratios that are consistent with proposed volumetric ratios of domain volumes (Figure S15, Supporting Information). Interestingly, more transient loops spanned a spectrum of states including a mix of exon/NE segments, suggesting stretched as well as packing configurations (Figure S15, Supporting Information).

**Figure 6:**
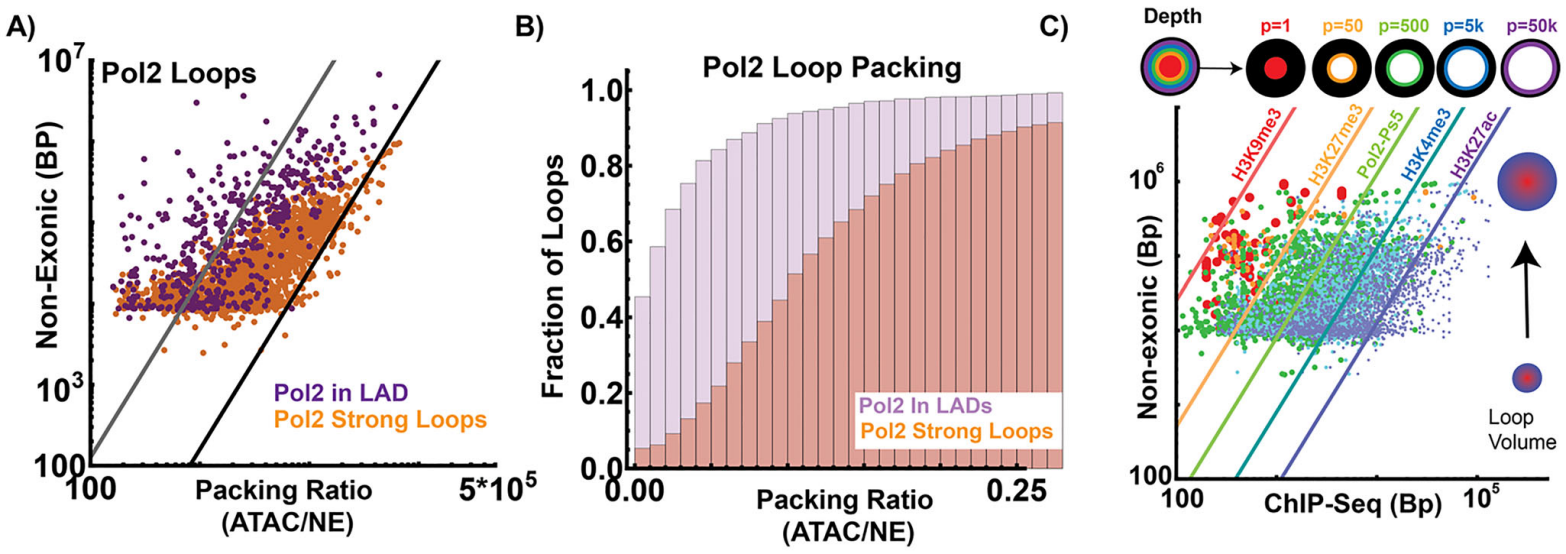
Genome geometry suggests transcriptional loops are efficiently packed. A) Analysis of experimentally observed Pol II transcriptional loops via ChiaPET in HCT‐116 cells. We observed that high‐frequency Pol II loops (>20 contacts) do not occur in lamin associated domains (LADs). Prior work has demonstrated that LADs are enriched in heterochromatin. Therefore, we compared the composition and packing ratio (ATAC‐Seq/NE) of strong loops in comparison to those observed in LADs. Consistent with transcriptional loops being packed through the nucleus, we observe packing ratios in both strong Pol‐II loops outside of LADs and within LADs. B) Analysis of the packing ratio of Pol‐2 loops in LADs demonstrates a lower packing ratio, consistent with more efficient packing this regions. C) Experimentally observed RNA polymerase loops plotted as a function of their NE content (approximately the volume generated) compared to the observed ChIP‐Seq content within each loop in HCT‐116 cells. Within large polymerase loop domains, we observe an accumulation of heterochromatin. The total heterochromatin content increases a function of the size suggesting packing occurring as a function of the volume generated. Small loops are primarily composed of euchromatin, indicating their volume would appear to be relatively decompacted. Collectively, this suggests that transcriptional loops have a degree of packing that correlates to the packing behavior for domain volumes observed on ChromSTEM imaging.

To test if stable loops are experimentally packed as a function their hypothesized size, we again utilized publicly available ATAC‐Seq and measured the observed packing compared to NE content. If loops are not packed (they exist as a chain), then accessibility would likely be a linear function of loop length as we detailed above. Consistent with the packing hypothesis, we observed that loop accessibility as measured by ATAC‐Seq, is a power‐law of NE length (**Figure** **6**a). This occurs even upon depletion of RAD21, indicating that the stable volumes are not dependent on cohesin extrusion for sustained maintenance. That RAD21 depletion did not significantly alter stable loop volumes was consistent with ChromSTEM imaging showing mature domains remain after RAD21 depletion^[^ ^12^, ^16^ ^]^ (Figure S14, Supporting Information). Interestingly, in contrast to accessibility in predicted volumes, accessibility in strong Pol‐II loops shifted towards deeper segments, suggesting that strong loops may be efficiently packed. Given that lamin associated domains (LADs) are regions enriched for heterochromatin (in the presented hypothesis, segments with lower S/V ratios), we next tested if transcriptional loops within LADs have similar packing properties to strong transcriptional loops throughout the nucleus. Analysis of the packing ratio using ATAC‐Seq demonstrates similar organization between LADs and high frequency loops; in both cases, the loops appear to be packed. Crucially, transcriptional loops in LADs are more tightly packed than those observed in the nuclear interior (Figure 6a,b).

Next, we used ChIP‐Seq to test the hypothesis that loops are packed in a manner like the domain volumes observed on ChromSTEM imaging. As described above, mature domain volumes in ChromSTEM tomography are composed of both high density (heterochromatin) and low density (euchromatin) into a single functional volume. If loops are similarly organized, we hypothesized that loops experimentally will be a mixture of heterochromatin (core), Pol‐II (ideal zone), and euchromatin (outer zone) as a function of the NE DNA volume. Using ChIP‐Seq data from HCT‐116 cells, we tested this hypothesis and observed that this organization is present within stable loops produced by Pol II (Figure 6c). Furthermore, as hypothesized by the observations in domain segments above, each functional layer of a domain is present as a function of the NE content to a different possible depth. Features that represent the core of domains (H3K9me3, H3K27me3, Figure 6c) were at shorter predicted depths (*p* ranging from 1 to 50 basepairs, Figure 5f), whereas Pol II aligned with theorized exon depths, and euchromatin was positioned to outer depths of the volume. Collectively, this indicated that transcriptionally mediated loops have features consistent with packing in a manner that resembles ChromSTEM imaging of packing domain volumes and the presented theory. Finally, it is worth noting that large loop segments contained heterochromatin at the deepest portions that are coupled to RNA polymerase II and euchromatin modifications that are distributed at depths consistent with the different functional zones proposed. When paired with the observation of Pol II loop packing ratios in LADs compared to the nuclear interior, it is consistent with the hypothesis of these structures being packed as domain volumes.

### 2.11 Geometry Suggests Packing Is Associated with Differentiation and Oncogenic Risk

We now explore the potential risks and benefits of the proposed geometric system hypothesis. The process of domain formation and maturation echoes that of reinforcement learning systems in artificial intelligence and neural networks.^[^ ^12^, ^66^, ^67^ ^]^ Inputs (signaling cascades, mechanical force) intersect with the current state (existing domains, ionic conditions, nuclear volume, and nucleosome remodeling complex concentration) that generate an output (RNA synthesis, domain activation/modification/degradation) that continuously co‐emerge in tandem. Signals that sustain the transcriptional output result in a competition for the local available space (volumetric DNA) to position exonic elements to the ideal zone shell. Since the generated output modifies the state (domain formation, reinforcement, or degradation), a future input signal acts in a different state that was generated by the prior inputs. The proposed geometric system organizes the human genome into several thousand domains guided by transcriptional inputs, the selection of which co‐emerges with the existing state of the cell from this competition. Collectively, it produces a system where coordinated behaviors across a tissue through shared reinforcement learning restricted by the available packing states. The domain geometric system, therefore, can encode a crucial feature for multicellular systems: a mechanism to produce coherent, reinforceable, and predictable memories of prior events (signals). This proposes an efficient, non‐mutational system to increase complexity since geometry (packing volumes) stores states defined by, and regulating, transcriptional reactions. If geometry can contribute to body plan complexity through alterations in volumes, genes active in terminally differentiated human tissues are expected to be primarily power‐law compositions, based on these considerations.

**Figure 7:**
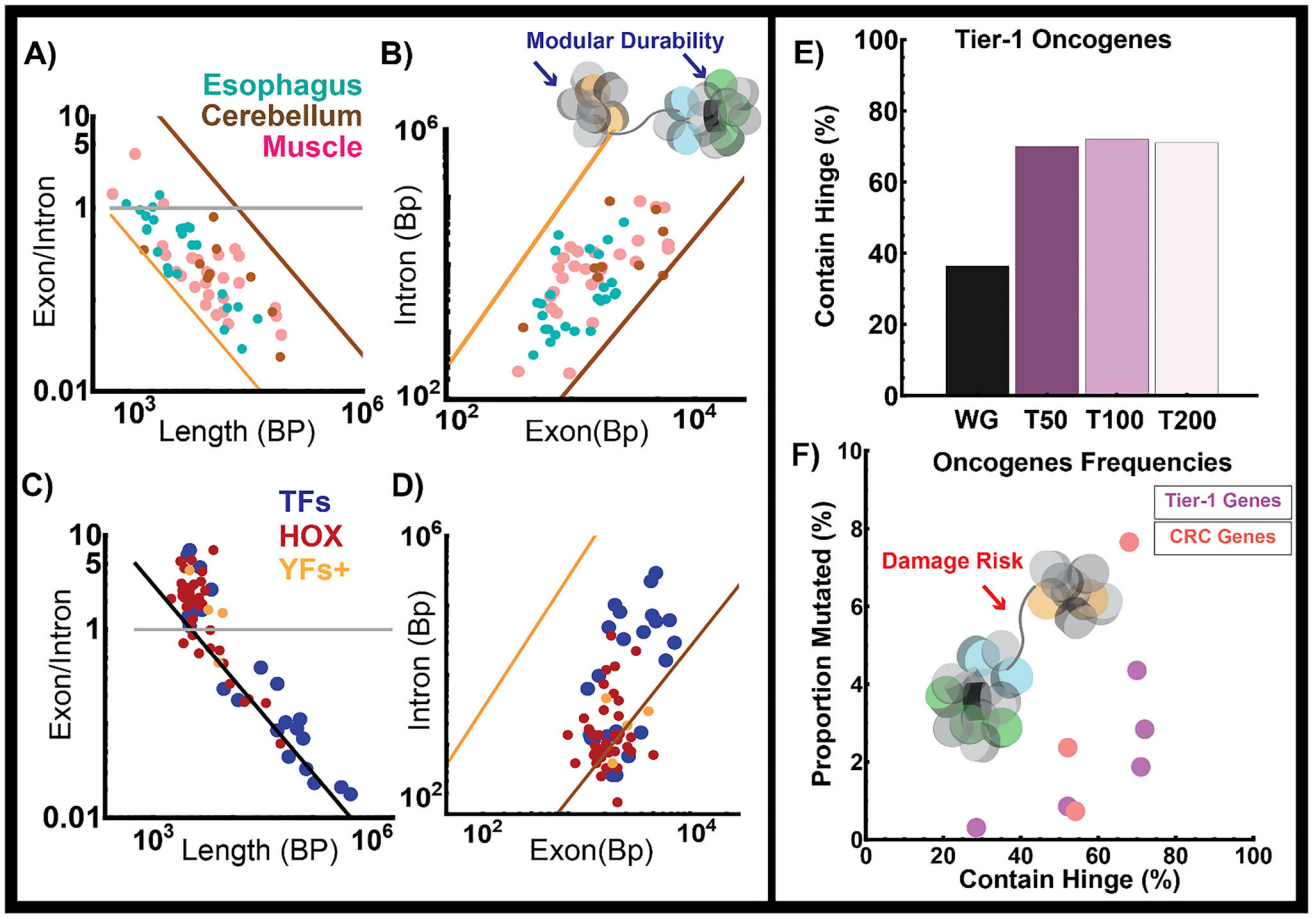
Power‐law geometry produces a trade‐off between modular durability and damage‐risk. A,B) Analysis of geometric properties of genes that are primarily unique to the esophagus, cerebellum, and muscle tissue demonstrating power‐law organization. C&D) Stem‐cell related transcription factors (Yamanaka factors, YF) generally organize as linear geometries. Similarly, HOX genes demonstrate two phenotypes: a cluster with linear organization (values of E/I >1) and a group that organizes into power‐law distribution. Transcription factors such as RUNX2 that define tissue function are primarily organized as power‐law geometries. E) Analysis of the frequency of protein‐coding genes containing a hinge position demonstrating that the 200 most frequent Tier‐1 oncogenes contain at least one hinge position. WG – whole genome compared Tier‐1 oncogenes by their frequency: T50 – top 50 genes, T100 – top 100 genes, T200 – top 200 genes. F) Analysis of Tier 1 oncogene frequencies demonstrates an acceleration, then plateau in frequencies as the likelihood of containing a hinge increases. Genes that are less likely to contain a hinge element had a lower correlation with oncogenic mutation frequency. Independent analysis of oncogene mutation frequencies in colorectal cancer (pink). This effect appears to plateau at mutation frequencies occurring over 1% of the time in both Tier‐1 oncogenes and colorectal cancer mutations.

To test if this is indeed observed, we analyzed genes involved in maintaining differentiated tissues from each germ layer: esophageal mucosa (endoderm), cardiac muscle (mesoderm), and cortical neurons (ectoderm). We utilized GTEx expression data from these three sites and selected genes that were preferentially associated with each tissue (Table S1, Supporting Information).^[^ ^15^ ^]^ We observed conservation of the domain geometry system for genes involved in maintaining tissue function across the human lifespan (**Figure** **7**a,b). Given this finding, we explored whether transcription factor families involved in differentiation and signaling processes were similarly organized. Embryogenic development is a complex process, with the timing of transcription factor activation impacting tissue formation. Therefore, we analyzed transcription factors in relation to developmental timing as a comparison. We found that genes of early development (pluripotency factors, HOX genes)^[^ ^68^, ^69^ ^]^ favored exon enrichment (linear structure or very small domains) whereas end‐organ factors (e.g., MITF^[^ ^70^ ^]^ or RUNX2^[^ ^71^ ^]^) contain packing ratios that are consistent with the formation of large, power‐law domains (Figure 7c,d, Table S2, Supporting Information).

However, by the proposed nature of a hinge guided by transcriptional activity, a portion of genes is placed in relatively risky conditions as they require being spanned between multiple reaction volumes. The risk to gene segments could arise from multiple possibilities, including replication timing, risk for entanglement events, exposure to free radicals, etc. As a result, our hypothesis predicts that these segments would be at a higher risk of mutations across all cancers. To test if this is the case, we investigated the mutation frequency of Tier‐1 oncogenes compared to the likelihood that these genes overlap with a hinge position. We utilized the reported mutation frequencies across all tumor types generated by ROSETTA, which analyzed frequencies from publicly available cancer genomics sources (i.e., TCGA/TARGET Program).^[^ ^72^, ^73^ ^]^ As these frequencies span across all tumor tissue types, they would provide an understanding of a conserved mechanism across cancers independent of tissue‐specific factors. The null hypothesis is that no association would be observed and that mutations would be independent of the generated geometry. Remarkably, we observed that the frequency of oncogenic mutations is strongly correlated with the likelihood of a gene containing a hinge position (Figure 7e,f). Indeed, many crucial Tier‐1 genes (both oncogenes and tumor suppressors) overlap with the presence of a hinge including TP53, BRCA1/2, ATM, RB1, IDH1/2, and PIK3CA (Table S3, Supporting Information). To validate these findings, we utilized colorectal cancer (CRC) as a representative model with the recently described landscape in over 2000 patients.^[^ ^74^ ^]^ We analyzed the mutation frequencies in all CRC patient samples compared to hinge overlap, observing similar correlations between hinge overlap and mutation frequencies (Figure 7f, Figure S16, Supporting Information). This property is observed in the primary tissue samples of both microsatellite instability and microsatellite stable CRC tumors. Indeed, mutations in microsatellite‐stable tumors were similarly enriched in hinge overlap (Figure S16, Supporting Information).

Interestingly, our results suggest some additional sequence specific factors may intersect with the proposed instability of hinge positions as they are relatively rich in GC content (Figure 3). Collectively, these findings suggest that the proposed geometry may produce a risk to genes that are required to create bifurcations between domains. While further work is necessary to understand if positioning is causal, it suggests that oncogenic mutation frequency may be linked to geometry. One could probe these properties in future work by potentially creating synthetic spacing elements in the context of genomic stress, measuring the relative rates of mutation, strand breaks, and the intersection with replication timing.

### 2.12 Packing Geometry Parallels the Emergence of Organo‐Axial Development

**Figure 8:**
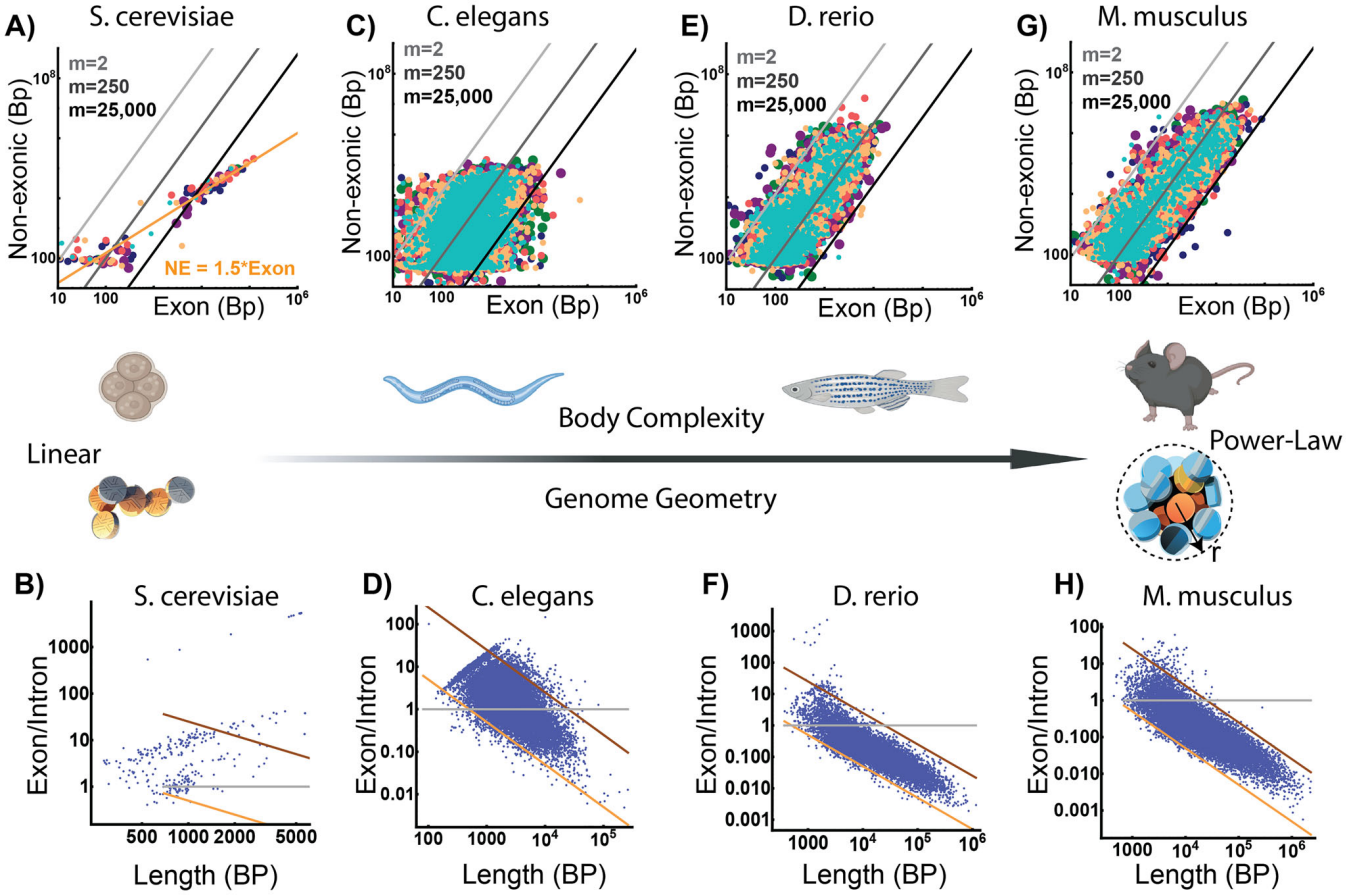
Packing geometry parallels body plan complexity in metazoans. A,B) Analysis of chromosomal architecture (A) and genes (B) demonstrates that the S. cerevisiae genome is most likely organized as linear beads on a string assembly. As gene length increases, the non‐exonic content increases linearly to create a chain. C,D) Analysis of chromosomal (C) and genes (D) demonstrating a transition toward power‐law assemblies. In C. elegans, genes appear to be equally split between linear assemblies (E/I >1) and power‐law assemblies. E–H) We observe a transformation with increasing body‐plan complexity in both genes and chromosomes of D. rerio (E,F) and M. musculus (G,H) that their organizational structure resembles the structure of genes and chromosomes observed in humans.

Since we propose that the benefit of the proposed geometry hypothesis is to produce durable cell states based on reinforcement learning, we conclude by performing a comparative analysis across eukaryotic genomes to understand if the proposed geometry is potential emergent mechanism. We specifically chose to analyze the following species due to their well‐characterized genomes and existence of nucleosomes, introns, intergenic segments, and splicing machinery: *S. cerevisiae* (a model of a monocellular organism with splicing and introns), *C. elegans* (a multi‐cellular organism with simple organoaxial positioning), *D. melanogaster* and *D. rerio* (multi‐cellular organisms with complex organoaxial positioning), and *M. musculus* (a non‐primate mammal with complex organoaxial positioning).^[^ ^75^ ^]^ If the proposed geometric system is present in all the investigated genomes to a comparable extent, it would suggest that geometry is independent of body‐plan complexity. Instead, if geometry correlates with increased development of durable cell states, it may present a mechanism to support the complexity of metazoan cell types. Consistent with the hypothesis that geometric encoding could facilitate organ complexity, power‐law coupling of exons with introns appears to parallel organ specification **Figure** **8**a–h). We observed the transformation from linear geometries with exon enrichment (*S. cerevisiae*) first toward a mix of power‐law and linear structures (*C. elegans*) (Figure 8a–h). As complexity increases, there is a further transition from an equal mix of linear and small geometric assemblies to primarily geometric assemblies in *D. rerio* onwards. Indeed, the genome of *M. musculus* presents a similar pattern of packing ratios observed within the human genome.

## 3 Discussion

We set out to understand how packing domain information is stored within the genome to explain the paradoxical decrease in accessibility of introns and intergenic segments for transcriptional activation during muscle differentiation (Figure 1). We arrived at the novel hypothesis that the positioning of exons, introns, intergenic segments could encode packing information to generate nanoscale volumes in a sequence‐independent manner. This proposes a crucial role for non‐exonic DNA to act as “volumetric DNA” in complex, multicellular eukaryotes to optimize chemical reactions involved in transcription.^[^ ^14^, ^49^, ^50^ ^]^ True mechanistic perturbations remain to be performed to validate this hypothesis and to fully prove the implications of the proposed theory. Nevertheless, this hypothesis introduces a mechanism for storing volumetric information that can optimize transcriptional reactions throughout the genome (Figure 1).^[^ ^9^, ^10^, ^38^, ^54^, ^58^, ^59^ ^]^

It is worth comparing this hypothesis to existing models of chromatin structure, including LLPS, loop extrusion, and hierarchical assembly. These results have elements resembling features of LLPS – exonic elements and euchromatin compositions appear as the interface between a domain volume and the outer zone region. This could support an evolutionary mechanism for “condensates” to form with a distribution of sizes. Instead of heterochromatin and euchromatin being antagonistic condensates, they are reflections of the size and packing efficiency of the packed domain. While not explored here, compartments observed on Hi‐C may intersect with the S/V ratios described. Indeed, recent modeling has suggested that this may occur.^[^ ^76^ ^]^ Larger domains have a tendency to contain more packed nucleosomes whereas smaller volumes have the inverse. This would lead to the perception of alternating segments with different contact ratios. This requires further investigation into the mechanisms of packing in the nucleolus and nuclear exterior as well as the behavior of CTCF loops in contrast to the Pol2 loops described herein. Interestingly, recent experimental evidence in live cells is consistent with S/V assembly of transcriptionally active regions of the genome constrained by cohesin.^[^ ^77^ ^]^

In contrast to an equilibrium process of LLPS, this hypothesis proposes that the act of transcription itself guides packing assembly. Therefore, instead of spontaneous demixing of molecules, the genome is guided by enzymatic processes resembling self‐assembly. As a result, loop extrusion may not be purely a topological barrier to heterochromatin expansion, but the mechanical act of transcription or extrusion on a short “hinge” segment may define the generated volumes. These assembled volumes, in turn, can interact with nucleosome remodeling enzymes to facilitate packing structures.^[^ ^16^ ^]^ Consistent with this are the observed enrichment of CTCF binding motifs and binding on hinge positions (Figure 4). It would be reasonable to consider that spacing produced from focal extrusion guides the self‐assembly process in concert with additional reactions from nucleosome remodelers. This would be consistent with recent observations in ChromSTEM imaging, where the depletion of RAD21 and inhibition of transcription impair nascent domain formation.^[^ ^12^, ^16^ ^]^

Limitations of the proposed hypothesis include the need to understand processes beyond transcription alone – including RNA splicing, DNA replication, and chromatin dynamics – intersect with packing geometry. We hope that future mechanistic studies guided by this hypothesis address these questions. For example, studying how chromatin packing intersects with splicing machinery could provide insights of how the rate of splicing can greatly exceed the rate of RNA synthesis of long introns.^[^ ^78^, ^79^, ^80^ ^]^ Interestingly, we observe on RIP‐Seq and EU‐Seq a length‐dependent decrease in Pol‐II bound RNA in long introns (Figure S14, Supporting Information), suggesting splicing efficiency may also be guided by geometry. In the context of replication, one could explore if replication stress and timing could be guided by geometric constraints. Although TAD differentially intersect with replication timing, prior work has shown that PDs are not TADs.^[^ ^16^, ^81^ ^]^ For example, one could explore how DNA polymerase size intersects with PD volumes and hinge positions. An interesting possibility is that replication timing is also guided by physical constraints, with heterochromatin rich regions being completed last due to S/V principles. In this example, one may study if replication starts near engaged “hinges” and proceeds through the proposed segments. Smaller segments with a higher S/V would be completed earlier whereas longer segments with a smaller S/V would take longer to complete. As the larger segments enrich for more heterochromatin, these would likely be completed towards the end of *S* phase. If these findings are observed, it suggests that packing could act as a system to store cellular transcriptional memory that is heritable across cells (**Table** **1**).

**Table 1:** Chromatin packing and physically organized reinforcement learning. Description of the properties of chromatin packing domains observed on ChromEM and their interactions with enzymes. The integration of packing domain elements with genomic spacing may allow future investigation into chromatin as a reinforcement learning system.

Terminology	Description	Simplified Schematic
**Packing Domain (PD**	The nanoscale physical structure of the genome measured on ChromEM imaging. These have a distribution of sizes and packing efficiencies related to their structure‐function	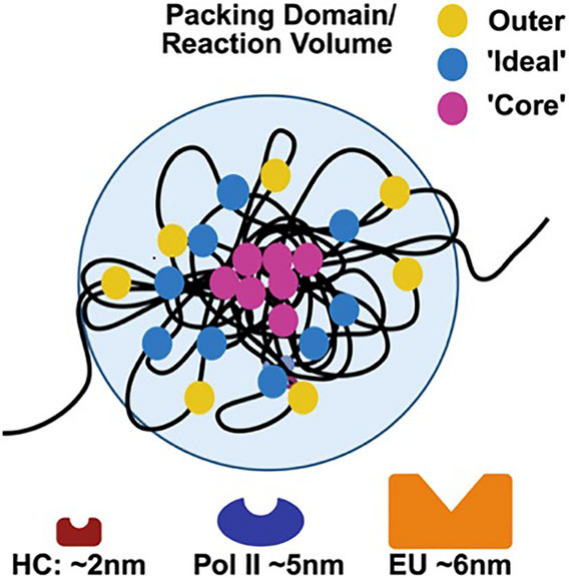
**Domain Core**	The high density interior of a packing domain. Mainly composed of heterochromatin (HC)	
**Ideal Zone**	Intermediate density shell where transcription reactions (Pol II) are hypothesized to be most efficient	
**Outer Zone**	Low density exterior of a packing domain. Mainly composed of euchromatin (EU)	
**Reaction Volume**	Description of packing domains from the perspective of the chemical reactions of chromatin remodeling enzymes, transcription factors, and polymerases. These proteins function concurrently in the generated PD space at different depths.	
**Fractal Dimension (*D*)**	A quantitative measure of the filling of a chromatin segment in 3‐D space	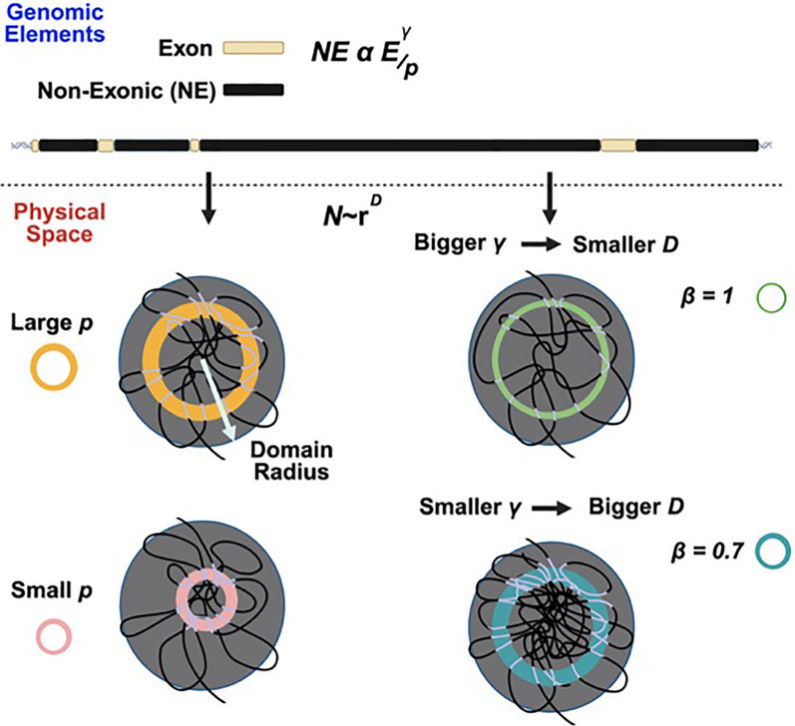
**Gamma** (γ)	A quantitative measure of the spacing of genomic elements. Theorized to describe filling in 3‐D reaction volume such that exons are positioned to the ideal zone shell.	
P	A quantitative measure of the depth of spacing of an element as it fills the 3‐D PD reaction volume.	
**Beta (ß)**	A quantitative measure of how the chromatin chain fills the 'ideal' zone shell within the total volume. ß of 1 is consistent with a hard, narrow shell whereas ß is consistent with no S/V relationship. The majority of the genome is consistent with a ß ‐0.7	
**Hinge segment**	A short genomic segment (1–300bp long, ‐1‐2 nucleosomes) composed of an exon with an adjacent non‐exon. The short size of these segments is theorized to create domain segments through the activity of RNA polymerase	
**Power‐Law Segment**	A genomic segment between two hinge positions that is composed of exons (shell) and non‐exons (volumetric). Theorized to be arranged as a shell‐to‐volume projection to optimize exon interactions with RNA polymerase	
**Self‐assembly**	Transcription guiding genomic elements to form into domains that have the optimal configuration for further transcriptional reactions. This is a process driven by the physical and chemical properties of domains	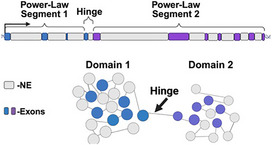
**Reinforcement Learning within Reaction Volumes**	A theorized form of machine learning dependent on physical and chemical properties of chromatin interacting with enzymes. Agents (e.g. enzymes like Pol II) interact with the state (chromatin polymer) within the existing state context (previously formed domains, ionic and redox conditions, physical space), executing chemical reactions (e.g. RNA synthesis, forced returns/jumps). The reward/penalty is the capacity to maximize domain formation and RNA synthesis.	
	Successful transcription is theorized to enhance domains. The result is that genetic elements are effectively competing for domain space. Instead of pre‐encoded guidance, chromatin‐enzyme interactions are emergent and context dependent. Converging on a form of physically encoded geometric computing.	

These considerations converge on the risks and benefits of the proposed geometric hypothesis to multicellular organisms. There are potentially immense benefits from the proposed system: space and information optimization, durability, efficiency, simultaneous enzymatic processing, and geometric modulation to increase degrees of freedom (Figures 1 and 2). In linear configurations (e.g., *S. cerevisiae*), activation of one gene could come at the expense of a neighbor becoming packed. Alternatively, transcription in linear genomes could occur in the span connecting domains, potentially indicating a mechanism by which the process progressed from a linear to a power‐law assembly. While a linear assembly has benefits for rapid simultaneous synthesis, it may lack the capacity to efficiently produce memory and specialization. In effect, compaction within *S. cerevisiae* effectively translates into a 3‐D barrier, whereas this is not the case in the assembly of domain volumes.^[^ ^82^ ^]^ Instead, genomes built on a volumetric geometry can produce complex, highly dynamic, and durable states. Complex bodies have many unique cell types that are adaptive to novel stimuli (infections, environmental stress, etc), store memory of the foundational state, and be able to effectively communicate coherently across different cell types. Geometry could introduce a system where information can be dynamically stored and retrieved, not just within DNA sequences and nucleosome modifications, but in the produced segment volumes. Since producing volumes guided by transcription a subset of genes that reside between domains, these may be at increased risk of mutations (Figures 7 and 8). With these considerations in mind, we describe several transformative hypotheses to test across different disciplines below.

### 3.1 Future Directions

#### 3.1.1 Hypothesis 1): Domain Geometry Facilitated the Rapid Emergence of Body Plan Diversity

The transformation of chromosomes from linear to power‐law assemblies parallels increasing body plan complexity (Figure 7). We naturally wonder if this converges at the time of the Cambrian Expansion^[^ ^83^, ^84^, ^85^ ^]^ as domain geometry can be a non‐mutational system to not only to increase degrees of freedom, but to produce memory and coherence across cell types. This occurs because changing configurations depends on modifying spacing and position without requiring sequence mutations. Cells could store information by generating packed volumes that guide transcription factor searching, gene availability, and loop end search times through their movement in 3‐D space instead of along the DNA chain. A volumetric organization system may solve a crucial problem in organisms with complex body plans, since they require a reliable communication system across all cell types that combinatorial search patterns through sequence searching may not efficiently achieve. This is because organs arise from the coordinated differentiation of many different cell types from a common progenitor; therefore, cells need to both stably retain memory of identity and reliably communicate with diverse but similar neighbors. This hypothesis can be tested by using the framework described here and then measuring the change in exon sequence composition compared to the change in arrangement. If evolutionary trajectories are found to be encoded in the arrangement of elements, it would demonstrate that selective pressure converges on gene position, orientation, and segment lengths to generate cohesive assemblies. What we perceive as “neutral drift” from the sequence perspective is potentially balanced by selection for maintaining volumes. This allows increased sequence sampling of these regions mutationally for potentially beneficial states while maintaining a “default” state as volumetric elements. Such findings would suggest that genomic selection is driven by the benefit of the system, and not necessarily the gene sequence alone.^[^ ^49^, ^50^ ^]^

#### 3.1.2 Hypothesis 2): Volume Stabilization of Mature Domains Defines Cell Response Across Decades

Nuclear swelling and heterochromatin loss are hallmarks of aging across human tissues.^[^ ^59^, ^86^, ^87^, ^88^ ^]^ Tissue development is defined by the non‐random deposition of heterochromatin.^[^ ^89^ ^]^ The volume ratios in packing domains reflect these states.^[^ ^54^, ^90^, ^91^ ^]^ With this in mind, several diseases may be influenced by domain degradation over time. For example, among the risk factors for Alzheimer's is the read‐through fusion of ApoE and Tomm40^[^ ^92^ ^]^ (Figure 1). Could nuclear expansion and heterochromatin loss shift reaction volumes to produce transcriptional “fusions” by positioning segments along the ideal zone as a single element^[^ ^93^ ^]^? Could a similar process be involved in inflammatory diseases since the misfolding of domains could create transcriptional memories that sustain inflammation^[^ ^94^ ^]^? These processes would naturally take a substantial amount of time to occur across the genome as geometry degrades. Finally, in cancer, chromosome fusions, fragmentation, and copy number variations are significant prognostic factors. We observed that oncogene mutation frequencies were correlated with localization to a hinge segment (Figure 6); could these be non‐random events that are related to how the structure responds to local conditions? In the context of domains facilitating body plan complexity, this process may represent a natural tradeoff in complex multicellular systems. While the organization of chromatin into domains facilitates transcriptional memory critical for complex body plans, it also exposes hinges to mutations, thus creating a conflict between advantages during the reproductive timespan of an organism and the accumulation of neoplastic mutations later in life. Indeed, mutation frequency of genes containing hinges is potentially mitigated by repair mechanisms resulting in a delayed risk of chromosomal abnormalities. The nanoscale and microscale transformation of the nuclear packing is similarly a hallmark of malignancy and chemoresistance. Increasing the total genomic content, shifting the positioning of genes, and altering nuclear volumes generate unexpected geometries and the loss of coherent responses to the same stimuli. If packing domains are built on the principles of reinforcement learning, it's possible that these considerations may extend to other diseases of aging. In much the same way that malignancy can result in distorted domain packing, cells could potentially hard‐code aberrant states during the aging process. Based on the principles of domain geometry, one could detect or even prevent these events by targeting the physiochemical conditions that define the structure of domains.^[^ ^74^, ^95^, ^96^ ^]^

#### 3.1.3 Hypothesis 3): Domain Geometry Accommodates Latency

Among the mysteries of transcriptional patterns is an unused surplus of binding sites for key transcription factors (e.g., Myod).^[^ ^97^, ^98^, ^99^ ^]^ While it has been suggested that these are errors, we can reconsider them in terms of information latency based on domains acting as a computational system. The default state generates volumes when unused, which remain as a reservoir for new domains if conditions change. In effect, the default positions needed for a human body plan are encoded in the measured positions of exonic/volumetric pairs with some degree of error to preserve a non‐default adaptation strategy. If these adaptations improve fitness, they could be hard‐coded for the next progeny. Repositioning of these elements and the deposition of transcription factor binding sites based on geometry could provide insight into how sequence specificity intersects with geometric specificity.

#### 3.1.4 Hypothesis 4): Chromatin Is a Geometric Computational System

By considering physical properties of the genome at the intersection with transcription reactions, these elements mirror aspects of learning, computation, and neural networks.^[^ ^66^, ^67^ ^]^ Some of the positional elements observed mirror elements of logic operators or computations, such as “AND” where genes in a paired segment become co‐transcribed and “OR” states, where one gene competes with another for volume. In this context, the predictable spacing between exons, introns, and intergenic segments requires inputs from the state of the cell. Alterations in mechanics,^[^ ^38^, ^100^ ^]^ ions,^[^ ^96^ ^]^ redox state,^[^ ^101^, ^102^ ^]^ and nucleosome remodeling enzymes^[^ ^59^ ^]^ can provide this information. Domains would then act as geometric processors, with the structures formed representing the intersection of inputs and the current state. While this is more challenging to test, manipulating gene positions or controlling the order of signals could produce insights into this process. If this were demonstrated experimentally, it may transform the understanding of chromatin from a passive instructional system into dynamic physical computational system. Finally, from the perspective of synthetic biology, controlling volume positioning during the generation of artificial chromosomes may guide the ability to generate complex traits.

Collectively, the presented geometric hypothesis requires additional mechanistic investigation for causality. The proposed system currently invites new avenues of exploration of genomics at the intersection of molecular, physical, chemical, and computational properties. Grounded in the intersection of these fields, exploration into the influence of domain volume assemblies on cellular fitness and organism evolution could serve as a framework for future studies in critical processes such as DNA replication, repair, and splicing.

## 4 Experimental Section

### Geometric Positioning of Exons and Volumetric DNA in Relation to Packing Domains

Scaling relationships are derived to assess whether non‐exonic (NE) DNA is coupled with exons to generate volume assemblies. Comparison between this analytical model and sequencing and ChromSTEM experimental data suggests that non‐exonic segments likely correspond to volumetric organization surrounded by exons behaving as a “wavy” line on the reaction zone of the volume provided by the non‐exonic elements, with domain fractal dimension *D* inversely related to γ.

Chromatin packing domains are nanoscopic, heterogeneous mass‐fractal structures. The transformation of a chromatin chain into volume is defined by how the length of a segment of the chromatin polymer within a 3D domain, M, scales as a function of the radial distance of the volume containing the polymer, *r* by $M∼r^{D}$, where *D* is the fractal dimension. The chromatin volume fraction, ϕ, at the radial distance *r* is defined by:

(5) $$\phi \;\left(r\right)=\phi_{0}{\left(\frac{r_{c}}{r}\right)}^{3-D}$$

where ϕ_0_ is the chromatin volume fraction at *r* = 0 (domain center) and *r_c_* is the chromatin chain radius.^[^ ^9^, ^10^, ^13^ ^]^ The total amount of chromatin in basepairs within the domain volume will therefore be:

(6) $$N\;\left(r\right)=N_{c}A_{D}{\left(\frac{r}{r_{c}}\right)}^{D}$$

with *N* being the basepairs contained within the radius, N_c_ the number of basepairs within the chain, and A_D_ the packing efficiency of the chain within a domain. Noting that A_D_ = 1 indicates efficient packing throughout the domain volume.

For hard 3D objects (e.g., a hard sphere), the number of basepairs, N_s_, within a zone shell of radius ΔR that is much smaller than the radius of the volume (ΔR << r) is:

(7) $$N_{s}\;\left(r\right)=\frac{dN}{dr}\Delta R$$

Substituting in Equation (6), therefore it was observed that:

(8) $$N_{s}\;\left(r\right)=D\times N_{c}A_{D}{\left(\frac{r}{r_{c}}\right)}^{D-1}\frac{\Delta R}{r_{c}}$$

Accounting for the fact that the chain elements are a polymer that can go in and out of the hard shell (e.g., the “wiggly” line in Figure 1), it is instead necessary to take the fractional derivative to capture the behavior at the reaction zone:

(9) $$N_{s}\;\left(r\right)=\frac{dN}{dr^{\beta }}\Delta R^{\beta }$$

where β is the order (dimension) of the derivative and ranges from 0 ≤ β ≤1.

Utilizing the chain rule for a function f(x):

(10) $$\frac{df}{dx^{\alpha }}=\frac{df}{dx}\;\frac{dx}{dx^{\alpha }}=\frac{1}{\alpha }\;x^{1-\alpha }\frac{df}{dx}$$

and Equations (9) and (10), therefore, it was observed that the composition of the ideal zone is:

(11) $$N_{s}\;\left(r\right)=\frac{1}{\beta }\;r^{1-\beta }\frac{dN}{dr}\Delta R^{\beta }=\frac{D}{\beta }\;N_{c}A_{D}{\left(\frac{r}{r_{c}}\right)}^{D-\beta }{\left(\frac{\Delta R}{r_{c}}\right)}^{\beta }$$

Assuming that the content of exons, E, in basepairs is approximately that of the contents of a domain ideal zone *E* ∝*N_s_* then the proportionality constant in this relationship is ∝β. From this, it was observed that:

(12) $$E\;\left(r\right)=DkN_{c}A_{D}{\left(\frac{r}{r_{c}}\right)}^{D-\beta }{\left(\frac{\Delta R}{r_{c}}\right)}^{\beta }$$

With *k* representing the fraction of the zone basepairs that are exons. Likewise, the length of a segment within a domain volume is the number of basepairs within that volume, *L*(r) = *N*(r). Transcriptional reactions occur at the ideal zone (the “Goldilocks zone”), the region where the balance between density stabilizes the intermediate complexes without overly limiting diffusivity of the reactant species.^[^ ^95^, ^96^, ^103^, ^104^ ^]^ For a domain limited by its ideal zone, the total length of the gene, L(r = R_gl_), and the exons, E(r = R_gl_) within domains to the goldilocks radius are:

(13) $$L=N_{c}\;A_{D}{\left(\frac{R_{gl}}{r_{c}}\right)}^{D}$$

which indicates that

(14) $$\left(\frac{R_{gl}}{r_{c}}\right)={\left(L{/}_{N_{c}A_{D}}\right)}^{1/D}$$

Solving for E using Equation (12) utilizing the relation from Equation (14) the exon contents were calculated by

(15) $$E=Dk{\left(N_{c}A_{D}\right)}^{\left(\beta /D\right)}\delta_{gl}L^{\left(\frac{D-\beta }{D}\right)}with\;\delta_{gl}={\left(\frac{\Delta R_{gl}}{r_{c}}\right)}^{\beta }$$

For simplicity, now let $Y\;=\;Dk{\left(N_{c}A_{D}\right)}^{\left(\beta /D\right)}\delta_{gl}$ and $C=\frac{D-\beta }{D}$ which simplifies Equation (14) to

(16) $$E=YL^{C}$$

with *C* generally bounded between 2/3 and 1 due to the limits of *D* in cells of 2 to 3.

The existence of power‐law scaling between exon length, E, and total gene length, L suggests a scaling relationship between the intron/intergenic segment and the exon. An exponent, γ in Equations. (2) and. (4) was observed such that $I=\frac{E^{\gamma }}{m}$.

If $\gamma =1+\frac{1}{C}$ then $I=\frac{E^{1+1/c}}{p}$, this would result in $\frac{I}{E}=\frac{E^{1/c}}{p}$ which by Equation (15) becomes $\frac{I}{E}=\frac{Y^{1/c}L}{p}$. Given that Y is nearly 1, this results in $\frac{I}{E}≅\frac{L^{n}}{p}$ consistent with the experimental observations in Equation (1). Translating the power‐law scaling described by γ into the mass‐fractal dimension of domains, it was observed that

(17) $$\gamma =1+\frac{1}{C}=1+\frac{D}{D-\beta }$$

as described in Equation (4).

For a domain that extends outside of its ideal zone, some NE regions may be found at r > R_gl_. Treatment similar to the one described above can be applied. From Equations (6) and (11), it follows that

(18) $$\begin{array}{ccc} & & L=N\left(R_{e}\right)=\frac{3}{D}\;N_{c}\varphi_{0}{\left(\frac{\varphi_{0}}{\varphi_{e}}\right)}^{\frac{D}{3-D}},\\ & & E=N\left(R_{e}\right)=3kN_{c}\varphi_{0}{\left(\frac{\varphi_{0}}{\varphi_{gl}}\right)}^{\frac{D-\beta }{3-D}}{\left(\frac{\Delta R}{r_{c}}\right)}^{\beta } \end{array}$$

where φ_*e*_ is the chromatin volume fraction at the radial distance corresponding to the outer bound of the domain, r = *R_e_* . In a special case of φ_0_ = φ_*gl*_ , manipulation of Equation (18) leads to

(19) $$E=kD{\left(\frac{3}{D}\varphi_{gl}\right)}^{\frac{\beta }{3}}{\left(\frac{\Delta R}{r_{c}}\right)}^{\beta }L^{1-\frac{\beta }{3}}$$

Again, a power‐law scaling relationship was seen among *I*, *E*, *L*:

(20) $$\frac{I}{E}\propto L^{n},\;I\propto E^{\gamma },\;\mathrm{and}\;E\propto L^{C}$$

Now the positioning of elements was considered depending on the property of the exon segments in relation to the ideal zone elements defined by β. For β = 1, the entire contents of the exon are within the hard shell. In contrast, for β = 0, the exon portion is volumetrically distributed indicating that there is no geometry.

Indeed, in the most likely case, where exons constitute a portion of the ideal zone (a wiggly line), then 0 ≤ β ≤1 with *D* of domains ranging experimentally on ChromSTEM and in polymer modeling between 2 and 3 (2 ≤ *D* ≤ 3).^[^ ^9^, ^10^, ^54^ ^]^ This results in 2 ≤ γ ≤ 3 observed experimentally.

When γ = 2 and β = 0, exons are distributed for any observed *D* (no geometric relationship). Conversely, when γ = 3 and β = 1, *D* = 2 results in the limiting case of a chromatin polymer in a good solvent. The most frequently experimentally observed γ ≈ 2.2–2.3 is achieved when β ≈ 0.6–0.7, indicating a substantial congruence between the ideal zone and exons, for experimentally (ChromSTEM) observed most probable *D* ≈ 2.8. It is worth noting that in this derivation, the observed γ and *p* values plotted are those of the ensemble, and not the realized values for each individual gene. In essence, the entire genome is organized by this behavior; however, each individual gene may require segments from a neighboring segment in order to achieve the realized domain.

### Power‐Law Genomic Analysis

RefSeq genomes were obtained from Igv.org/app mapping to the UCSC genome browser assemblies. The selected genome assemblies were as follows: Human (GrCH38), Mouse (GRCm39), Zebrafish (GRCZ11), Drosophila melanogaster (dm6), and S. cerevisiae (sacCer3). These text files were converted to xlsx extensions and then imported into Mathematica v14 for subsequent analysis with custom‐built code. Genes were then separated to analyze protein coding with the assigned prefix “NM” and non‐protein coding “NR”. The first isoform was selected for analysis of individual genes and compared to the analysis with all isoforms. At the level of chromosome analysis, all isoforms were considered and projected onto the chromosome for positions of exons and introns. A separate analysis of the first isoform was performed with similar findings. Exon start and stop positions were used for segmentation, and an reciprocal start/stop position for introns was generated. For simplicity, all exons were assumed to be part of the gene within the isoform variant. For chromosome wide analysis to account for the direction of transcriptional reading frames, genes were separated by their location to the positive or negative strand orientation for analysis. Multi‐start or multi‐stop exon overlapping events accounted for less than 7% of exon positions but were omitted in whole chromosome analysis for simplicity. Hinge elements were subsequently identified either for the whole chromosome in the read orientation (positive, negative) by mapping in the orientation of the read‐frame (exon‐NT) such that an exon with an adjacent non‐exon was below the reported threshold: ≈1–300 bp (1–2 nucleosome hinge). For simplicity, a single threshold was used in this study unless otherwise reported of 300 bp or less. The length of transcribed (exonic) and non‐exonic (NE) sequences was summed in the intervening segments. For these analyses, all chromosomes (1‐22, X‐, and Y‐) were included. No regions were omitted based on GC content, lack of genes, or other features. Sequence alignment was performed on GrCH38.14 for mapping of DNA sequence content to hinges of 300 bp size. The equations above were compared as described for the observed exon/intron ratio versus gene length, exon versus intron, and exonic versus NE in the respective figures. Randomization of only exons occurred by utilizing the generated lengths of exons and randomly repositioning these segments throughout the length of a chromosome. The remaining space was then defined as non‐exonic. Randomization of an exon with its associated intron occurred by random permutation of the position of the shared elements along the generated lists for each chromosome. This randomization process resulted in producing chromosome lengths within 5% of those reported in GrCH38 with ≈97% of the exonic content retained for both controls.

Randomized comparison for features enriched to hinges compared to non‐hinges was performed by controlling for the number of hinges observed on that chromosome and the maximum size specified (300 bp). This was compared to randomly sampling the same number of hinges and produced similar results; therefore, for simplicity, the same number of hinges was used for control purposes. Overlap between enhancers, nucleosome modifications, and specific proteins was performed such that any portion of the hinge or the control segment was at least partially covered.

In brief, each position of exons, introns, and intergenic segments was obtained from RefSeq for the respective genomes analyzed. It was hypothesized that the genome was composed of a distribution of power‐law segments interspaced by short segments (hinges). Genes were defined by their start‐stop positions, and exons by the respective coordinates in each RefSeq annotation. The non‐exonic segments were then identified as the interval gaps between exon coordinates within genes (introns) and between genes (intergenic segments). Each chromosome was then segmented into pairs of start and stop values for each element {{{Exon1_start, Exon1_end}, {NE1_start, N1_end}}, …, {ExonM_start, ExonM_end},{NEM_start, NEM_end}} where *M* constitutes the last position. A separate paired table of lengths was generated by {{Exon1_stop – Exon1_start, NE1_stop – NE1_start}, … {ExonM_stop – ExonM_start, NEM_stop – NEM_start}}. Utilizing the inbuilt Mathematica function, *SplitBy[]*, a series of nested tables were created such that the chromosome was split whenever a “hinge” position was observed Total[{{Exon1_stop – Exon1_start, NE1_stop – NE1_start}]≤300. The total exon content and non‐exon content were calculated in each segment within the generated table and the values were plotted as shown in Figures 4 and 7, and 8. This results in a nested tables for each chromosome in each reading frame orientation of {{Exon_content1, NE_content1}, {{Exon_content1, NE_content1}, …, {Exon_contentM, NE_contentM}}. Then the positions of individual power‐law segments were retrieved. The position of hinges was similarly identified by the complementary function, Position[], searching for the positions where the total length was less than 300 bp. These were stored as indexed tables for each chromosome of their start and stop coordinates.

For hinge overlap, the coordinates above for each chromosome were utilized. Search through the respective files (.bed and.bedpe files) was conducted for statistically significant peak start/stop coordinates for the nucleosome modifications. Tables of the hinges with marker overlap of at least 1 bp were then generated for each chromosome and the frequency of overlap was calculated relative to the total number of hinges in that chromosome. As a control, an equal number of randomly distributed positions that are 300 bp long on each chromosome were generated. As with the hinges, the likelihood of overlap was calculated with the markers such that at least 1 bp matched the track. The frequency of overlap was then calculated and plotted for each individual chromosome as presented in the figures. Statistical testing using the inbuilt T‐test function in Mathematica was performed on the generated distribution of events per chromosome.

For sequence composition analysis, GRCh38.p14_genomic.fna build was utilized. The respective coordinates obtained above were used to obtain the sequence composition within the hinge positions and exons for each chromosome. Then the GC content in each coordinate was measured and the overall frequency per chromosome was calculated. To identify specific sequence motifs, the StringTake command was utilized and searched for the CTCF core motif (CCCTC) in the respective hinges as well as the reverse complement GAGGG. The frequencies were then compared to those in any individual exon. T‐tests were then performed on the per‐chromosome frequencies observed.

To analyze the composition of segments in between hinges (Pol‐II, H3K9me3, etc) with segments between hinges, first hinges that were engaged with Pol‐II Ps5 as these are theorized to be “engaged” were identified to demarcate segments based on the presented theory. Then the content of each mark within the generated segment in the positive and negative orientation was calculated, plotting the contents against the total non‐exonic elements in the interval power‐law segment.

The R^2^ for each plot was calculated as follows. The fraction of unexplained variance was utilized to calculate the R^2^, R^2^ = 1‐ (Unexplained Variance)/(Total Variance). Owing to the fact that the distribution of Exon/Intron ratios spans from 10^3^ to 10^−3^, the R^2^ in these cases was calculated by measuring the log‐10 residuals. The unexplained and total variance was analyzed in the log‐10 distribution. Statistical testing was performed by measuring the residuals of the model against the random control in comparison to the observed ratios in the human genome. For analysis of R^2^ for non‐exonic versus exonic values, the residuals were calculated in Cartesian space.

Utilizing the Tissue Dashboard through the GTEx Portal, genes from the top 50 expressed within each respective tissue were subselected to represent different embryonic origins such that they generally did not overlap with other tissue beds. The selected tissues were the cortex (ectoderm), cardiac muscle (endoderm), and esophagus (endoderm).

### ChromSTEM Analysis

In a 3D ChromSTEM tomogram, domains are analyzed by first identifying the domain centers and then identifying the properties of the genome within the domain volume as previously described.^[^ ^9^, ^10^ ^]^ In brief, domain centers are identified as 2D local maxima of a 2D projection map along the imaging axis, followed by a Gaussian filter (5‐pixel radius) and local contrast enhancement (CLAHE) using ImageJ (Fiji).

For each domain, the 2D correlation of mass versus distance away from the center is calculated. The calculation is repeated and averaged using a 11 × 11 pixel window in all the slices of the 3D tomogram for the final correlation $M\;\left(r\right)=\frac{\sum_{i}m_{i}\left(r\right)\times w_{i}}{\sum_{i}w_{i}}$, where *w_i_* is the mass of chromatin at center voxel i, and *m_i_* (*r*) is the mass correlation function obtained using voxel i as the center. Domain boundary is defined as a change of the slope from the mass correlation function by using the MATLAB ischange function.

After the calculation, the mass correlation of each domain can be described as $M\;\left(r\right)=A\times {\left(\frac{R_{domain}}{R_{min}}\right)}^{D}$, with packing efficiency A, radius of domain *R_domain_* and DNA chain size *R_min_*. The estimation of genomic size of the domain is by $\mathrm{Genomic}\;\mathrm{Size}\;=A\times {\left(\frac{R_{domain}}{2}\right)}^{D}\times 15\;\mathrm{bp},$ where 15 bp is the estimated size of crystalline DNA in a 2 nm cube volume and assumed to be equivalent to the brightest possible 2 nm cube voxel in the whole tomogram.

### EU‐RNA‐Seq for Nascent RNA Analysis–Sample Generation

Nascent RNA was labelled, captured, and sequenced following the protocol reported by Palozola et al.^[^ ^105^ ^]^ In brief, to selectively label nascent RNA, HCT116 cells were treated with media containing 0.5 mM 5‐ethynyluridine (EU) for 1 h (Click‐iT nascent RNA Capture Kit cat. no. c10365, Thermo Fisher Scientific). These RNAs were retrieved using Click‐iT chemistry to bind biotin azide to the ethylene group of EU‐labeled RNA. The EU‐labeled nascent RNA was purified using MyOne Streptavidin T1 magnetic beads. Captured EU‐RNA attached to streptavidin beads was immediately subjected to on‐bead sequencing library generation using the Universal Plus Total RNA‐Seq with NuQuant (Tecan) according to the manufacturer's protocols with modifications. On‐bead complementary DNA (cDNA) was synthesized by reverse transcriptase using random hexamer primers. The cDNA fragments were then blunt‐ended through an end‐repair reaction, followed by dA‐tailing. Subsequently, specific double‐stranded barcoded adapters were ligated, and library amplification for 15 cycles was performed. PCR libraries were cleaned up, measured on an Agilent Bioanalyzer using the DNA1000 assay, pooled at equal concentrations, and sequenced on a Novogene Nova Seq X with 50 BP paired end. Two independent replicates were generated and pooled.

### Data Analysis

Paired end nascent RNA transcripts were trimmed with TrimGalore v0.6.10 and aligned using Hisat2 v2.1.0. Alignment files were sorted, filtered, and converted to BAM format using Samtools v1.6. Coverage files were generated using Deeptools v3.1.1 and normalized using RPGC normalization. Finally, aligned filtered reads were counted using separate genome annotation files for intron, exon, gene bodies, and intergenic regions with Htseq v2.0.2. Gene body coverage plots were generated using the Superintronic package described in Lee et al^[^ ^106^ ^]^ by binning each gene body into 20 bins in the 5′→3′ orientation and calculating the average coverage in each bin for short, average, and long genes within introns and on exons.

### Estimation of Compaction from Nucleosome “Beads on a String Arrays” Compared to Observed Sizes

Consider the hypothesis that genes are arranged as “open” 10 nm beads‐on‐a‐string configurations to facilitate the RNA synthesis from a gene body in its entirety. One can calculate the length of the nucleosome array for thyroglobulin gene (TG) and Titan (TTN) compared to experimental observations as follows.^[^ ^44^ ^]^ TG is ≈268 000 bp and TTN is ≈304 000 which converts to an array of 1340 and 1520 nucleosomes for 200 bp increments, respectively. Using the diameter of nucleosomes as 11 nm, this produces chains that are 14.74 and 16.72 microns long for each gene. The reported median inter‐flank distance in transcriptionally active state for each gene was observed as 703 and 1104 nm, respectively.^[^ ^44^ ^]^ This tenfold difference in length was accounted for by the introduction of stiffness, indicating the need for selective compaction of these genes even in their active state. A similar observation is observed within mouse neuronal cells in Rbfox1 (≈1.527 Mbp in mice, ≈2.4 Mbp in humans). In mice, Rbfox1 has a calculated chain length of ≈84 microns (200 bp/bead) as “beads on a string” but an experimentally observed transcription start site (TSS) to transcription end distance (TES) of ≈1 micron.^[^ ^107^ ^]^

This indicates an 84‐fold compression between the TSS‐TES during active RNA synthesis. A series of reaction volumes does not mean a gene does not undergo decompaction for transcriptional activation. Instead, it indicates that decompaction likely transitions from large domains into a series of smaller domains. The smaller reaction volumes could be predictably encoded by the spatial positioning between exons, introns, and intergenic segments to create efficient reaction volumes.

### Chromatin Connectivity, Enhancer, Epigenetic Modifications, and Tier‐1 Oncogenes

The respective data of Chromatin interaction analysis with paired end tag (ChiaPET), Chromatin Immunoprecipitation with Sequencing (ChIP‐Seq), and RNA‐Sequencing Analysis (RNA‐Seq) were all obtained from ENCODE.^[^ ^17^, ^18^, ^19^ ^]^ The.bed and.bedpe files were downloaded locally from replicate conditions in the reported table, and in‐built Mathematica software was used for analysis. The data for lamin associated domains was obtained from the 4D nucleome.^[^ ^108^, ^109^ ^]^ The respective bed and bedpe files used are uploaded and listed in Table S3 (Supporting Information). The analysis for the composition of ChiaPET loops was described above, where the total content in both orientations was considered for each chromosome. For analysis of element overlap with hinge positions, the identified 300 bp hinges were used, and the likelihood of any portion overlapping with a histone modification peak or enhancer peak was calculated. As a control variation in the read lengths covered by each element, the hinges observed when exons were randomly scrambled across the chromosome length were used. A two‐tailed t‐test comparing the observed frequency of overlapping of the feature with a hinge per chromosome was compared to the observed frequency in the hinges of randomly generated segments.

RefSeq gene positions used to define the start/stop position of gene annotations as described in Almassalha et al.^[^ ^59^ ^]^ Using the.bed files from ENCODE as above, the mean location of each peak was identified for the different marks with a p‐value cut‐off of at least <0.1. For each cell line, the Pol II‐PS5 peaks was organized by its density within each gene, ranging from at least 1 to 10. Then the distance of the Pol II‐PS5 peak to the nearest peak of the respective histone mark was calculated. Using the cumulative density of distance, the median distance was calculated as a function of the number of Pol II‐Ps5 peaks on a gene body. With respect to the per chromosome analysis, the total segment length of each mark was calculated for every somatic chromosome. Then the difference in chromosome length was normalized and the coverage of Pol II‐PS5 was plotted against each respective chromatin mark. In the case of human tissues, the association between heterochromatin and euchromatin was plotted since active RNA polymerase data were not available.

Analysis of oncogene mutational frequency compared to hinge positioning was performed by restricting the hinge length to 300 bp or less (≈1‐2 nucleosomes). The position of the hinges was mapped to protein coding genes. Then the fraction of genes from a Tier‐1 oncogene was calculated from the data within Mendiratta et al.^[^ ^72^ ^]^ Then the oncogenes into ascending groups were grouped and the mean mutational frequency observed for each group was calculated. The values were then plotted compared to the observed frequency of hinges within genes within that group.

### Multi‐Color Single Molecule Localization Microscopy–Cell culture

HCT116 cells (ATCC, #CCL‐247) were cultured in McCoy's 5A Modified Medium (Thermo Fisher Scientific, #16600‐082, Waltham, MA). The cell media were supplemented with 10% fetal bovine serum (FBS; Thermo Fisher Scientific, #16000‐044, Waltham, MA) and 100 µg/mL penicillin‐streptomycin (Thermo Fisher Scientific, #15140‐122, Waltham, MA). Cells were cultured under standard conditions at 37 °C in a humidified atmosphere with 5% CO2. Following detachment by trypsinization, cells were allowed to re‐adhere and recover for at least 24 h before further handling. Imaging was conducted when cell surface confluence ranged between 40% and 70%. For this study, cells were used between passages 5 and 20.

### Dual‐Color SMLM for EdU and Histone Modification–Cell Preparation and Fixation

After 48 h from being seeded, the cells were incubated with EdU for 2 h, followed by fixation for 10 min at room temperature with 4% paraformaldehyde in PBS. Fixed samples were washed three times in PBS for 5 min each. Secondary staining with AF647 via click‐reaction chemistry was then performed according to the manufacturer's protocol (ThermoFisher).

### Permeabilization and Primary Antibody Staining

Samples were permeabilized and blocked using a buffer containing 3% bovine serum albumin (BSA) and 0.5% Triton X‐100 in PBS for 1 h. They were then incubated with rabbit anti‐H3K9me3 or mouse anti‐H3K27me3/H3k4me3 (Abcam) diluted in blocking buffer for 1–2 h at room temperature on a shaker. Samples were washed three times in a washing buffer composed of 0.2% BSA and 0.1% Triton X‐100 in PBS.

### Secondary Antibody Staining and Imaging

The samples were incubated with goat anti‐rabbit or goat anti‐mouse AF488 (ThermoFisher) for 40–60 min at room temperature on a shaker. After incubation, samples were washed twice in PBS for 5 min each. Imaging was performed immediately, acquiring 10 000 frames per channel for each cell.

### Dual‐Color SMLM for EdU and BrdU

After 48 h of seeding, the cells were incubated with EdU and BrdU simultaneously for 2 h. This was followed by fixation for 10 min at room temperature using 4% paraformaldehyde in PBS. The fixed samples were then washed three times with PBS, each wash lasting 5 min. BrdU and EdU staining was performed according to the BrdU and EdU double staining protocol provided by Thermofisher.

### SMLM Reconstruction

The raw SMLM images were reconstructed using the built‐in Thunder‐STORM plugin in ImageJ. The camera setup parameters for the plugin were tailored to the imaging configuration. In the setup, a pixel size of 110 nm (calculated as the camera pixel pitch divided by the objective magnification), photoelectrons per A/D count of 1.09, and a base level of 0 were used. The peak intensity threshold coefficient was adjusted based on the acquisition quality, typically ranging from 1 to 2.

### Chromosome Paint

Human myoblasts were differentiated into myoblasts, and chromosome painting was performed on myotubes as described previously.^[^ ^37^, ^46^ ^]^ Images were acquired on a Nikon Confocal Microscope or a Zeiss LSM 800 Confocal Microscope. Imaging was done with a Plan Apo VC 100 × 1.4 NA oil objective as a multidimensional z‐stack. The acquired 3D image stacks were then fed through imaging processing pipelines utilizing standard tools on Cell Profiler and Fiji pipelines performed the functions of translating images to maximal projections, calculating distance, object size, nuclear size, and radius.^[^ ^110^, ^111^ ^]^

### RIP‐seq

HCT116‐POLR2A‐AID2 cells were treated with DMSO at a concentration less than 0.1% v/v for 5 h. Following DMSO treatment, protein‐bound RNAs were immunoprecipitated using the EZ‐Magna Nuclear RIP (Native) kit (Cat. # 17–10521) as follows. Cells were spun down and resuspended in ice‐cold resuspension buffer, homogenized, and lysed. Lysates were treated with DNase I. Inputs for the samples were withdrawn at this point. Lysates were incubated with 5 µg of protein A/G Magnetic Beads bound primary antibody for RNA Polymerase II Serine 2 Phosphorylated (ab252855, purchased from Abcam) and incubated on a rotating rack at 4 °C overnight. Following immunoprecipitation, lysates were again treated with DNase I before going through a wash step to eliminate DNA residue. After the wash steps, RNA was purified from IPs using Trizol reagent and chloroform per the protocol. Purified RNAs were processed as a library using the same library generation kit that was used for the bulk RNA‐sequencing. rDNA was removed for all samples except for one sample from each condition set aside for rDNA quantification. The Illumina HiSeq 4000 NGS System was used to generate single 50‐base reads for each sample.

### RIP‐seq Data Analysis

Paired end nascent RNA transcripts were trimmed with TrimGalore v0.6.10 and aligned using Hisat2 v2.1.0 to human reference genome GRCh38 (Ensembl) or to a custom hg38 reference that includes a 45 KB rDNA repeat for mapping to ribosomal DNA.^[^ ^112^ ^]^ Duplicate reads were marked and removed using Picard v.2.21.4.^[^ ^113^ ^]^ Alignment files were sorted, filtered, and converted to BAM format using Samtools v1.6. Coverage files were generated using Deeptools v3.1.1 and normalized using RPGC or BPM normalization. BAMs were merged for final consensus peak calling. Peaks were called on BAMs with MACS2 v. 2.2.9.1^[^ ^114^ ^]^ using the ‐q 0.1 –keep‐dup all parameters and with Pirhana (Uren 2013). Pirhana peaks were called using a q value cutoff of 0.1 and a binsize of 200 BP. Finally, aligned filtered reads were counted using separate genome annotation files for intron, exon, gene bodies, and intergenic regions with Htseq v2.0.2 with the Homo_sapiens.GRCh38.111.gtf (Ensembl) genome annotation. Genome annotations for introns and exons were generated using the methods described by Alkallas & Najafabadi (Alkallas & Najafabadi 2022). An intergenic genome annotation was generated using a custom script by subtracting the union of exons and introns from gene bodies and dividing the remainder into countable bins of 25 kb each. Gene body coverage plots were generated using the Superintronic package described by binning each gene body into 20 bins in the 5′→3′ orientation and calculating the average coverage in each bin for short, average, and long genes within introns and on exons. Matrices of peak coverage profiles were generated using Deeptools v.3.1.1 computeMatrix, and heatmaps were plotted using plotHeatmap.^[^ ^106^ ^]^ Differential gene expression analysis was performed using DESeq2 v.1.44.0 in R. Input versus coverage plots were generated by taking the overlap of coverage signal and RIP‐seq peaks called with MACS2 in the treatment condition. Intron‐centric differential expression analysis was performed using the Intron Differences To Exon or INDEX package (https://github.com/Shians/index) and Superintronic package (https://github.com/sa‐lee/superintronic) in R.^[^ ^106^, ^115^ ^]^ Visualizations of coverage files were generated using the Gviz package in R.^[^ ^115^ ^]^

### Declaration of Generative AI and AI‐Assisted Technologies in the Writing Process

During the preparation of this work, the author(s) used ChatGPT in order to ensure readability. After using this tool/service, the author(s) reviewed and edited the content as needed and took(s) full responsibility for the content of the publication.

### Data Sharing Plan

The generated analysis code and data will be made available upon publication. Public sources of data are included in the summary tables for reference.

## Conflict of Interest

The authors declare no conflict of interest.

## Author Contributions

L.M.A. and K.L.M. contributed equally to this work. Conceptualization was carried out by L.M.A., K.L.M., M.C., C.D., I.S., and V.B. The original draft of the manuscript was written by L.M.A. and K.L.M. Review and editing of the manuscript were performed by L.M.A., K.L.M., M.C., C.D., R.G., J.I., L.M.C., W.S.L., R.N., P.S.D., I.S., and V.B. Methodology was developed by L.M.A., R.G., I.S., and V.B. Resources were provided by L.M.A., K.L.M., P.S.D., I.S., and V.B. Funding acquisition was managed by L.M.A., K.L.M., P.S.D., I.S., and V.B. Supervision was provided by I.S. and V.B. Project administration was conducted by I.S. and V.B. Investigation was performed by L.M.A., K.L.M., R.G., J.I., L.M.C., and W.S.L. Validation was carried out by L.M.A. Formal analysis was conducted by L.M.A. and V.B. Visualization was created by L.M.A. and K.L.M. Software was developed by L.M.A. Data curation was performed by L.M.A.

## Supporting information



Supporting Information

Supplemental Table 1

Supplemental Table 2

Supplemental Table 3

Supplemental Table 4

Supplemental Table 5

## Data Availability

The generated analysis code and data will be made available upon publication. Public sources of data are included in the summary tables for reference.
